# FBP1 regulates proliferation, metastasis, and chemoresistance by participating in C-MYC/STAT3 signaling axis in ovarian cancer

**DOI:** 10.1038/s41388-021-01957-5

**Published:** 2021-08-06

**Authors:** Haoran Li, Zihao Qi, Yongdong Niu, Yufei Yang, Mengjiao Li, Yangyang Pang, Mingming Liu, Xi Cheng, Midie Xu, Ziliang Wang

**Affiliations:** 1 Cancer Institute and Department of Gynecological Oncology, Fudan University Shanghai Cancer Center, Shanghai, China; 2 Department of Oncology, Shanghai Medical College, Fudan University, Shanghai, China; 3 Department of General Surgery, Shanghai First People’s Hospital, Shanghai Jiaotong Univeristy School of Medicine, Shanghai, China; 4 Department of Pharmacology, Shantou University Medical College, Shantou, China; 5 Clinical Medicine Transformation Center and Office of Academic Research, Shanghai Hospital of Traditional Chinese Medicine Affiliated to Shanghai University of Traditional Chinese Medicine, Shanghai, China; 6 Shanghai Xuhui Central Hospital, Zhongshan-Xuhui Hospital, Fudan University, Shanghai, China; 7 School of Pharmacy, Anhui Medical University, Hefei, China; 8 Department of Pathology and Biobank, Fudan University Shanghai Cancer Center, Shanghai, China; 9 Department of Pathology, Fudan University Shanghai Cancer Center, Shanghai, China

**Keywords:** Cancer metabolism, Gynaecological cancer

## Abstract

Fructose-1,6-bisphosphatase (FBP1) is a rate-limiting enzyme in gluconeogenesis and an important tumor suppressor in human malignancies. Here, we aimed to investigate the expression profile of FBP1 in ovarian cancer, the molecular mechanisms that regulate FBP1 expression and to examine how the FBP1 regulatory axis contributes to tumorigenesis and progression in ovarian cancer. We showed that FBP1 expression was significantly decreased in ovarian cancer tissues compared with normal ovarian tissues, and low-FBP1 expression predicted poor prognosis in patients with ovarian cancer. The enhanced expression of FBP1 in ovarian cancer cell lines suppressed proliferation and 2-D/3-D invasion, reduced aerobic glycolysis, and sensitized cancer cells to cisplatin-induced apoptosis. Moreover, DNA methylation and C-MYC binding at the promoter inhibited FBP1 expression. Furthermore, through physical interactions with signal transducer and activator of transcription 3 (STAT3), FBP1 suppressed nuclear translocation of STAT3 and exerted its non-metabolic enzymatic activity to induce the dysfunction of STAT3. Thus, our study suggests that FBP1 may be a valuable prognostic predictor for ovarian cancer. C-MYC-dependent downregulation of FBP1 acted as a tumor suppressor via modulating STAT3, and the C-MYC/FBP1/STAT3 axis could be a therapeutic target.

## Introduction

Epithelial ovarian carcinoma is the most malignant tumor of the female reproductive system. Despite recent advances in epithelial ovarian carcinoma detection and treatment, the overall prognosis remains poor [1]. Therefore, there is still a need for the development of diagnostic and predictive molecular biomarkers to better understand the disease [2].

Tumor cells have a higher rate of aerobic glycolysis than oxidative phosphorylation [3–5]. FBP1, the rate-limiting enzyme in glycolysis, catalyzes the hydrolysis of fructose-1,6-bisphosphate (F-1,6-BP) to fructose-6-phosphate and inorganic phosphate. F-1,6-BP is a known allosteric activator of Pyruvate kinase isozyme type M2 (PKM2), which is an important enzyme in glycolysis. Therefore, FBP1 may inhibit the effect of glycolysis in tumor cells [6, 7]. Recently, low expression of FBP1 is regarded as a potential prognostic factor for malignancies including gastric cancer, breast cancer, and lung cancer [8, 9].

Downregulation of FBP1 expression causes an increase in glycolysis and the number of cancer stem cells (CSCs) [10]. Moreover, beyond its role in inhibition of glycolysis, some studies have reported that overexpressed FBP1 may directly suppress tumor growth and migration in breast cancer [10–12] and renal cell carcinoma by interacting with the hypoxia-inducible factor (HIF) domain [13]. It was also reported that FBP1 inhibited *ERK* activation and bypassed gemcitabine resistance in pancreatic cancer by blocking the interaction between IQ motif-containing GTPase activating protein 1 (*IQGAP1*) and *MAPK* [14]. Increasing evidence showed that low expression of FBP1 was caused by the methylation of FBP1 promoter [10, 15–17]. All these studies suggest that epigenetic regulation of FBP1 plays a critical role in modulating tumor initiation and progression in various cancer types.

In the present study, we validated FBP1 as a negative regulator of tumor invasiveness and chemoresistance in ovarian cancer. Mechanistically, we demonstrated that the loss of FBP1 expression in ovarian cancer was due to C-MYC-mediated promoter hypermethylation and that enhanced FBP1 directly interacted with STAT3 to inhibit its nuclear translocation and the subsequent activation of STAT3-regulated genes. Our results demonstrate the regulatory function of C-MYC/FBP1/STAT3 signaling axis on cell proliferation, metastasis, and chemoresistance in ovarian cancer.

## Results

### FBP1 expression is inversely correlated with tumor progression in ovarian cancer

**Fig. 1:**
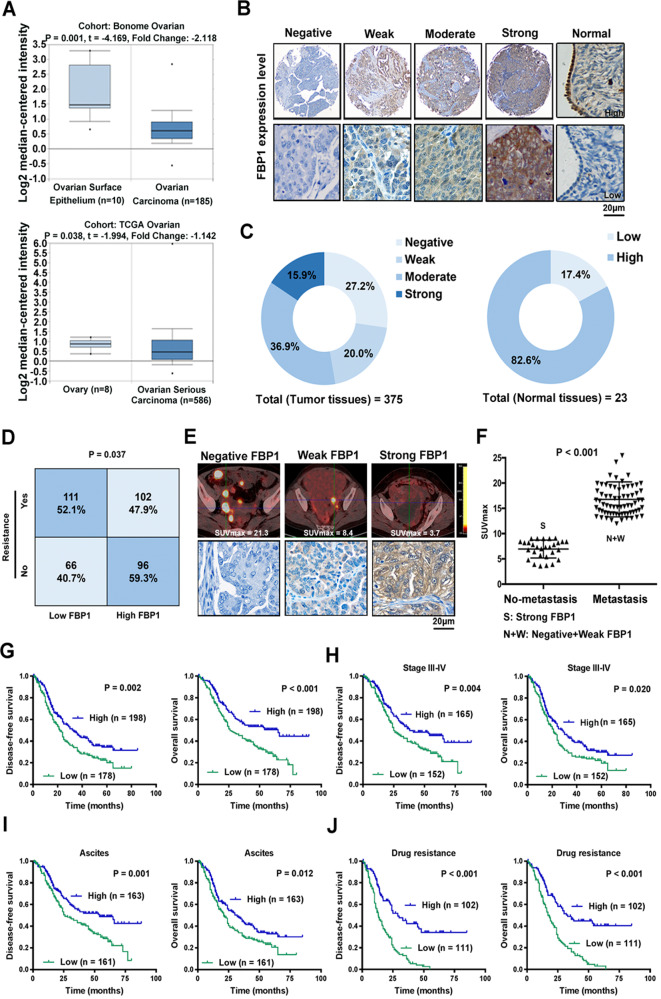
FBP1 inhibits the nuclear translocation of STAT3 via an enzymatic activity-dependent mechanism. **A** Box plots comparing FBP1 mRNA expression levels in the Bonome cohort and TCGA cohort were download from the Oncomine database. **B** Representative images from ovarian cancer and normal ovarian biopsy samples with various levels of FBP1 immunohistochemical staining (4× and 400×). **C** FBP1 expression in human ovarian carcinoma sections (*n* = 375, left) and normal ovarian tissues (*n* = 23, right). **D** Relationship of FBP1 expression with ovarian cancer chemotherapeutic resistance (*χ*^2^ test). **E** Representative ^18^F-FDG PET/CT images from ovarian cancer patients with negative (left), weak (middle), or high (right) FBP1 expression (all 400×). **F** SUVmax of ovarian cancer patients with low-FBP1 expression and metastasis or high-FBP1 expression and no metastasis (*n* = 100). **G** Kaplan–Meier DFS and OS curves (log-rank tests) for patients with high and low expression of FBP1 based on IHC staining scores. **H** Kaplan–Meier DFS and OS curves for patients with high or low-FBP1 expression levels in patients stratified by the tumor stage. **I** Kaplan–Meier DFS and OS curves for patients with high or low-FBP1 expression levels stratified by present of ascites. **J** Kaplan–Meier DFS and OS curves for patients with high or low-FBP1 expression levels stratified by present of drug resistance status.

To assess the clinical significance of FBP1 in ovarian cancer, we compared FBP1 mRNA expression in ovarian cancer and healthy ovarian tissue using data from the Oncomine database (www.oncomine.org). The FBP1 expression level was significantly lower in ovarian cancer tissue than in normal ovarian tissue in both the Bonome and TCGA cohorts **(**Fig. 1A). We then analyzed FBP1 protein expression in 375 tumor tissues and 23 normal ovarian tissues (FUSCC cohort) by immunohistochemical staining. We found that 47.2% (*n* = 177) of the tumor tissues exhibited high-FBP1 immunostaining (defined as moderate or strong), and 52.8% (*n* = 198) exhibited low-FBP1 immunostaining (defined as negative or weak) (Fig. 1B, C). FBP1 protein expression levels were high in a larger percentage of normal tissues (19/23, 82.6%) than tumor tissues (Fig. 1C). Analysis of the correlation of the FBP1 levels (grouped as high or low) with clinicopathological data showed that low-FBP1 expression was negatively associated with ascites, residual tumor size, the chemotherapeutic response, and recurrence (Supplementary Table 1). Moreover, we found that significantly more patients with high-FBP1 expression were sensitive to chemotherapy rather than resistant to chemotherapy in the FUSCC cohort (Fig. 1D). We then studied whether there was a correlation between FBP1 protein levels, metastasis, and SUV values using preoperative PET/CT scan data and immunohistochemical staining data from 100 ovarian carcinoma patients. We found that patients with no metastasis had high-FBP1 protein levels, whereas patients with metastasis presented with low-FBP1 levels; in addition, SUVmax values were significantly lower in patients with high-FBP1 protein levels than in those with low-FBP1 protein staining (Fig. 1E, F). These data indicate that FBP1 is downregulated and correlated with the tumor progression, metastasis, and chemosensitivity in ovarian cancer.

To evaluate if FBP1 expression could be used as a prognosis factor in ovarian cancer. We also evaluated the correlation between FBP1 immunostaining and the prognosis of ovarian cancer patients by survival analysis with the log-rank test. Patients with low-FBP1 expression had significantly shorter disease-free survival (DFS) (*P* = 0.002) and overall survival (OS) (*P* < 0.001, Fig. 1G) than patients with high-FBP1 expression. Further stratified analyses with different clinicopathological factors showed that among patients with an advanced tumor stage, the presence of ascites, or the presence of chemotherapeutic resistance, patients with low-FBP1 expression showed decreased DFS and OS compared with patients with high-FBP1 expression (Fig. 1H–J). Low-FBP1 mRNA expression was also found to be correlated with significantly worse OS (*n* = 1656, *P* = 2.5e−05) and DFS (*n* = 1435, *P* = 7.3e−05, Supplementary Fig. 1A) in an independent cohort available in the KMplot database (http://kmplot.com) [18], and further stratified analyses showed the same results among patients with advanced tumor stage (Supplementary Fig. 1B). Multivariate analysis with the Cox proportional hazards model revealed that chemotherapeutic response (HR 3.840, *P* = 0.000), FBP1 expression (HR 0.582, *P* = 0.000), and FIGO stage (HR 1.402, *P* = 0.028) were independent prognostic factors for OS in ovarian cancer (Supplementary Table 1). In addition, chemotherapeutic response (HR 2.481, *P* = 0.000), FBP1 expression (HR 0.621, *P* = 0.000), and FIGO stage (HR 1.693, *P* = 0.009) were independent prognostic factors for DFS in ovarian cancer. These data indicate that FBP1 expression positively correlates with patient prognosis in ovarian cancer, especially in patients at advanced stage, with ascites and chemoresistance.

### Modulation of FBP1 levels affects expression of genes involved in migration, proliferation, and chemosensitivity

Immunoblotting analysis of the background expression level of FBP1 in 12 ovarian cancer cell lines showed that the expression level of FBP1 was lower in A2780 and SKOV3 cells (Supplementary Fig. 2A) and higher in OVCA433 and OVCA420, so these four cell lines were selected for further experiments. We induced FBP1 cDNA in A2780 and SKOV3 cells and constructed stably FBP1-overexpressing cell lines (A2780/FBP1OE and SKOV3/FBP1OE cells, Supplementary Fig. 2B).

To explore the potential regulatory function of FBP1 in ovarian cancer, we used gene-chip assays to compare the expression of FBP1-related genes in A2780/FBP1OE cells with that in control cells. Through this approach, we found that genes involved in metastasis, apoptosis, response to cisplatin, and oxidative phosphorylation were enriched in cells with high-FBP1 expression (Supplementary Fig. 2C, D). All these signs indicated that FBP1 may play a critical role in ovarian cancer cell proliferation, apoptosis, metastasis, and response to cisplatin through involvement in oxidative phosphorylation.

### FBP1 inhibits proliferation, metastasis, and glycolysis in ovarian cancer cells

To determine the role of FBP1 in regulating cell proliferation, we performed CCK-8 and colony-formation assays and found that, compared with control cells, enhanced FBP1 suppressed cell growth and reduced the number and size of the colonies in A2780 and SKOV3 cell lines (Supplementary Fig. 3A–C). We then tested the effect of FBP1 expression on migration and invasion by transwell and scratch assay, and found that overexpression of FBP1 decreased the migration and invasiveness of A2780 and SKOV3 cells (Supplementary Fig. 3D–G). Western blotting results also demonstrated that FBP1-overexpressing reduced expression levels of MMP3 and Bcl-2, while increased E-cadherin of in A2780 and SKOV3 cells (Supplementary Fig. 3H). On the basis of these results, FBP1 appears to halt ovarian cancer cell proliferation, invasion, and migration, possibly by suppressing the expression of MMP3, and Bcl-2, and stimulating the expression of E-cadherin, which was consistent with the results of Zang et al. [19] in prostate cancer. To further validate the role of FBP1 in carcinogenesis, we silenced the expression of FBP1 in OVCA420 and OVCA433 cells (OVCA420/ShFBP1 and OVCA433/ShFBP1, Supplementary Fig. 4A). The colony formation assay (Supplementary Fig. 4B, C) and transwell assay (Supplementary Fig. 4D, E) showed consistent results as above, which indicating that FBP1 had a vital impact on the metastatic ability of ovarian cancer cells, which was consistent with our gene-chip results.

Since FBP1 is the rate-limiting enzyme in gluconeogenesis, we suspected whether FBP1 negatively regulates cell growth by blocking glucose metabolism in ovarian cancer cells. As shown in Supplementary Fig. 5A–E, glucose uptake, lactate and ATP production, extracellular acidification rate, and oxygen consumption rate were all dramatically decreased in cells overexpressing FBP1 compared with controls (*P* < 0.05). The detection of western blotting showed that enhanced FBP1 reduced expression levels of GLUT1, HK2, and LDHA in A2780 and SKOV3 cells (Supplementary Fig. 5F). In addition, PET-CT analysis showed that overexpression of FBP1 significantly suppressed the glucose uptake of xenografted ovarian cancer cells in vivo and resulted in a lower SUVmax value (Supplementary Fig. 5G).

### FBP1 sensitizes ovarian cancer cells to cisplatin and represses cell sphere-forming capacity of ovarian cancer cells

**Fig. 2:**
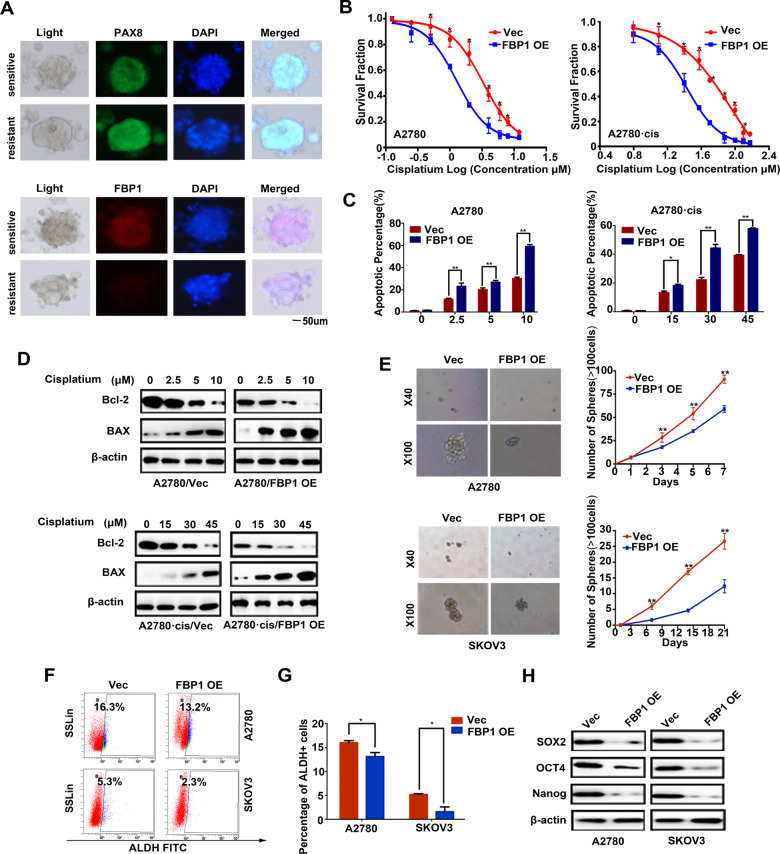
FBP1 overexpression sensitizes ovarian cancer cells to cisplatin. **A** IF staining of the representative human ovarian cancer organoid lines with PAX8, FBP1, and DAPI (DNA) as indicated. **B** CCK-8 assays showed the effect of empty vector and FBP1 OE on the chemosensitivity of ovarian cancer cells to the cytotoxic effect of cisplatin. **C** The percentage of apoptotic cells in indicated group after cisplatin treatment. Cells were stained with annexin V–fluorescein isothiocyanate (FITC) and propidium iodide (PI) to detect cells in early apoptosis (annexin V+ PI–) and late apoptosis (annexin V+ PI+). Representative pictures are shown. **D** The expression levels of Bcl-2 and BAX in vector control (Vec) and FBP1 overexpressing (FBP1 OE) ovarian cancer cells were examined by western blotting. **E** Representative images showed the representative number of spheres counted each day over a period of 7 days for A2780 cells stably expressing empty vector (Vec) or FBP1 (FBP1 OE). **F**, **G** The percentage of ALDH-positive ovarian cancer cells in the vector and FBP1 OE groups were determined by flow cytometry and statistically analyzed. **H** Immunoblotting analysis of the apoptosis-associated proteins SOX2, OCT4 and NANOG. Data are shown as the means ± SD. *P < 0.05, **P < 0.01.

To illustrate the role of FBP1 in ovarian cancer cisplatin resistance, we established 33 cisplatin-sensitive ovarian cancer organoids and 24 cisplatin-resistant ovarian cancer organoids (Fig. 2A). We found that the expression of PAX8, a marker of ovarian cancer, was higher in cisplatin-resistant ovarian cancer organoids than those with cisplatin sensitivity, but FBP1 was opposite **(**Fig. 2A). And then, we treated A2780 cells and the cisplatin-resistant ovarian cancer cell line A2780/DDP (A2780·cis) with different concentrations of cisplatin for 48 h. CCK-8 assays and flow cytometry analysis revealed that FBP1 increased the sensitivity to cisplatin of A2780 and A2780·cis cells (Fig. 2B, C). The loss of FBP1 decreased the sensitivity to cisplatin of OVCA420 and OVCA433 cells as well (Supplementary Fig. 6). Then, we studied the induction of apoptosis after 48 h of treatment with different concentrations of cisplatin, we found that overexpression of FBP1 led to the upregulation of the pro-apoptotic protein BAX but downregulated the anti-apoptotic protein Bcl-2 in a dose-dependent manner in both cell lines (Fig. 2D). Previous studies disclosed that CSC-like property was close correlated with chemotherapy resistance in cancer cells. To identify whether FBP1 affected CSC-like property of ovarian cancer cells, we performed a sphere formation assay and analyzed the proportion of ALDH expression. Compared with the control group, sphere formation efficiency was suppressed in both A2780 and SKOV3 cell lines with elevated FBP1 expression (Fig. 2E) and the proportion of ALDH^+^ expression was lower in cells overexpressing FBP1 (Fig. 2F,G). Consistently, FBP1 overexpression obviously inhibited the expression of SOX2, OCT4, and NANOG (Fig. 2H), which regulated CSC-like property in various cancers. Taken together, these results suggested that FBP1-overexpressing cells had a higher rate of apoptosis than control cells in response to cisplatin treatment, and that FBP1 overexpression synergized with cisplatin to inhibit cell proliferation, migration, and invasion.

### FBP1 inhibits the progression of ovarian cancer and sensitizes cancer cells to cisplatin-induced apoptosis in vivo

**Fig. 3:**
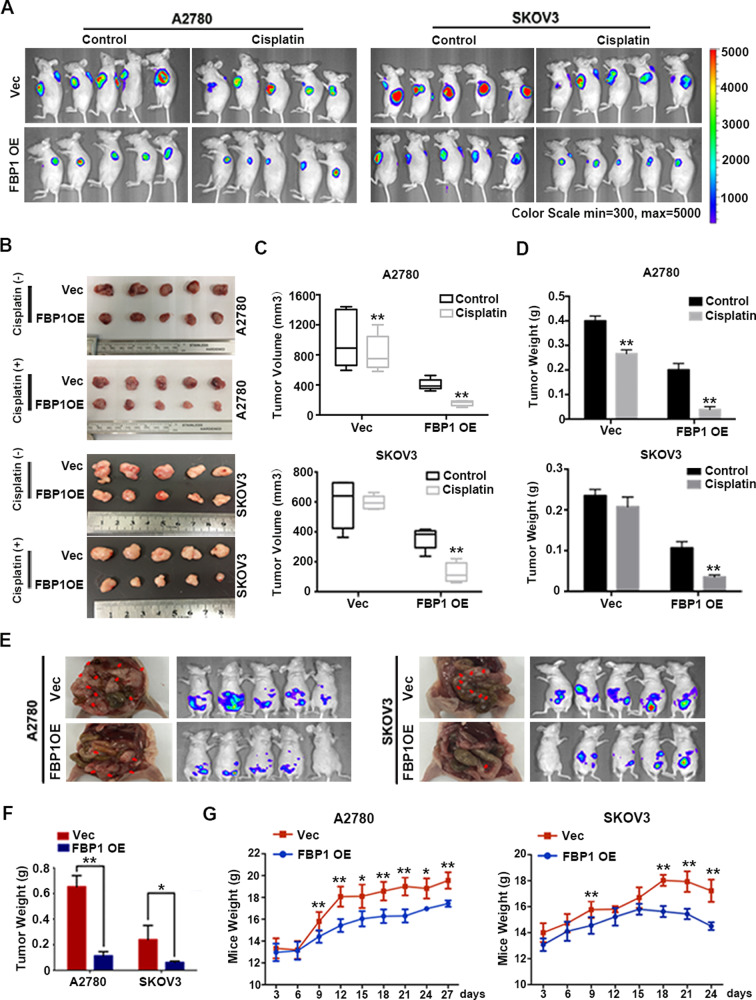
Influence of FBP1 on the progression and chemosensitivity of ovarian cancer in vivo. **A** Representative luciferase image of nude mice bearing tumors formed by FBP1*-*overexpressing A2780 or SKOV3 cells or transfected with empty vector (Vec) or FBP1 (FBP1 OE) (*n* = 5). **B** Representative image of nude mice bearing tumors formed by A2780 and SKOV3 cells. **C** The average tumor volume after cisplatin treatment. **D** The average tumor weight after cisplatin treatment. **E** Gross image (left panels) and representative luciferase image (right panels) of the intraperitoneal dissemination of tumors formed by A2780 and SKOV3 cells expressing empty vector (Vec) or FBP1 (FBP1 OE). Red arrows show the position of tumors in the nude mice. **F** The average tumor weight after intraperitoneal injection. **G** Average body weight of mice after intraperitoneal injection. Error bars = 95% CIs. *P < 0.05, **P < 0.01.

We next tested anti-tumor effects of FBP1 in vivo. To observe subcutaneous tumor formation, we injected A2780 and SKOV3 cells either overexpressing FBP1 or harboring empty vector into the flanks of nude mice. As is shown in Fig. 3A–D, overexpression of FBP1 slowed the speed of tumor growth and reduced overall tumor weight in vivo. After the tumor volume reached 100 mm^3^, the mice were treated with cisplatin on alternate days. Fluorescence imaging showed that the FBP1 overexpression led to reduced tumor volume and weight following cisplatin treatment, relative to cells harboring empty vector (Fig. 3A–D).

Next, using murine intraperitoneal xenotransplantation models, we found that overexpression of FBP1 inhibited tumor metastasis, reducing both the number and the weight of peritoneal disseminated lesions (Fig. 3E, F). Moreover, the weight of these mice was significantly less than controls (Fig. 3G). Our in vivo findings indicated that the overexpression of FBP1 could exert an anti-tumor effect on ovarian cancer cells and sensitize ovarian cancer cells to cisplatin treatment both in vitro and in vivo.

To determine whether FBP1 could decrease the expression of genes involved in epithelial–mesenchymal transition (EMT), apoptosis, and stemness, we performed IHC assay using transplanted murine tumors. Our results were in accordance with earlier results obtained in cell lines (Supplementary Fig. 7).

### The anti-tumor effect of FBP1 may be achieved by direct influence on the STAT3 expression

To further elucidate the molecular mechanism underlying the anti-tumor effect of FBP1, we performed mass spectrum analysis in A2780 cells with high expression of FBP1. The FBP1-bound complex was then analyzed using SDS-PAGE, and the gel was stained with Coomassie blue. The lane with the FBP1-bound complex was excised and subjected to LC/MS analysis, which identified STAT3 as a potential FBP1 binding partner (Supplementary Fig. 8A).

**Fig. 4:**
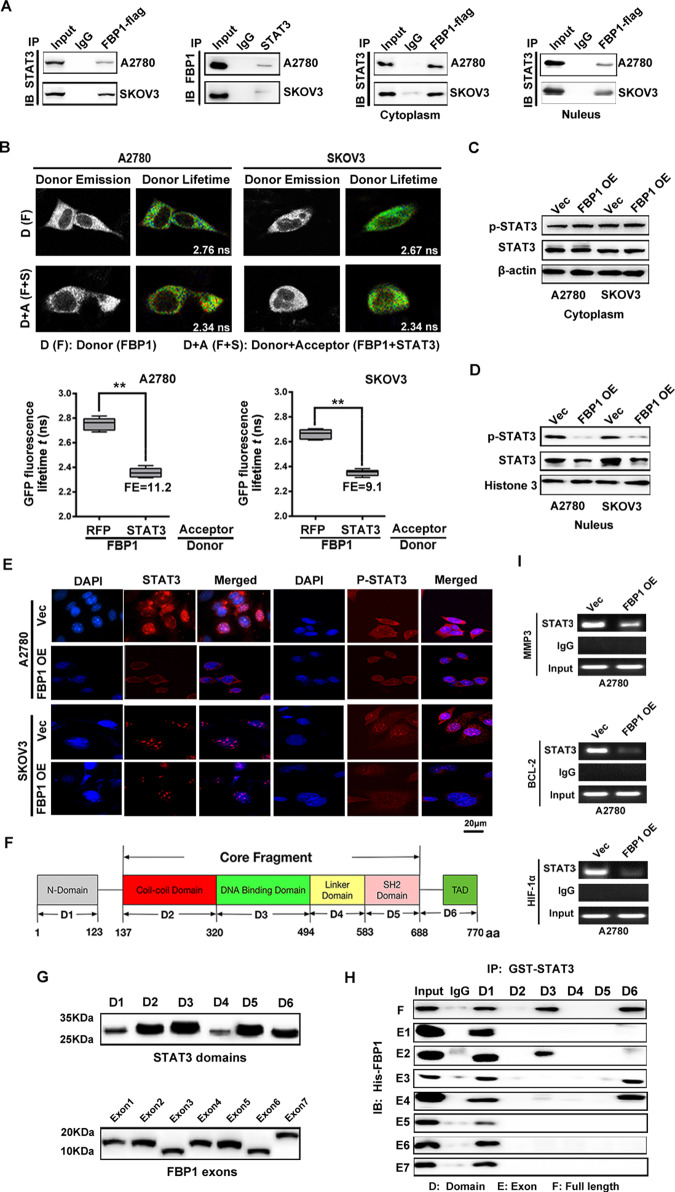
FBP1 directly interacts with STATS and inhibits STAT3 expression and phosphorylation in ovarian cancer cells. **A** Interaction between FBP1 and STAT3 detected by co-immunoprecipitation assay. **B** Interaction between FBP1 and STAT3 by FRET-FLIM upon transient coexpression in A2780 and SKOV3 cells. FE, FRET efficiency. Asterisks indicate a statistically significant difference (**P value < 0.01), according to the Student’s *t* test. **C**, **D** The influence of induction of FBP1 on the distribution and expression of STAT3 and p-STAT3 (Tyr705) protein in the nucleus and cytoplasm. **E** Representative immunofluorescence staining (×1000) images showing that FBP1 inhibited the expression of STAT3 in the cell nucleus (red). Blue dye (DAPI) indicates the nucleus. **F** Schematic image of functional domains of STAT3. **G** Western blot analysis indicated proteins ectopically expressed six regions of STAT3 and seven exons of FBP1. **H** Co-immunoprecipitation analysis indicated the interaction between STAT3 domains and FBP1 exons. **I** RT-PCR results of ChIP. The experiments were conducted in triplicate, and a representative experiment is shown.

The results of co-immunoprecipitation (Fig. 4A) and Fӧrster resonance energy transfer–fluorescence lifetime imaging (FRET-FLIM) (Fig. 4B) further demonstrated the interaction between FBP1 and STAT3, which confirmed the results analyzed from mass spectrometry assay.

We also found that overexpression of FBP1 decreased the expression level of STAT3 in A2780 and SKOV3 cells (Supplementary Fig. 8B), which was supported by the results of IHC assay using xenografted tumors (Supplementary Fig. 8C). Interestingly, we found that FBP1 overexpression significantly reduced the accumulation of STAT3 protein in the nucleus, whereas had no impact on cytoplasmic levels of STAT3 detected by co-immunoprecipitation and immunofluorescence assay (Fig. 4C–E). We thus hypothesized that FBP1 might regulate STAT3 by preventing nuclear entry, thereby blocking its ability to regulate the expression of downstream target genes. To explore this further, we cloned six functional domains of the STAT3 protein, including the N-terminal region (1–137 bp, D1), the coiled-coil domain (CCD, 137-320 bp, D2), the DNA-binding domain (DBD, 320-494 bp, D3), the linker domain (495-583 bp, D4), the SH2 domain (584–688 bp, D5) and the C-terminal region (transactivation domain, TAD, 689–770 bp, D6) (Fig. 4F–G). These were then expressed in vitro and subjected to a GST pull-down assay, which revealed that endogenous FBP1 protein (extracted from A2780/FBP1OE cell lysates) bound specifically to the fragments corresponding to D1 (1–137), D3 (320–494), and D6 (689–770) domains of STAT3 (Fig. 4H). To further map regions of FBP1 that are critical for STAT3 recognition, we ectopically expressed seven exons of FBP1 (Fig. 4G) and performed co-immunoprecipitation with epitope-tagged STAT3 protein domains (Fig. 4F). We found that STAT3 D1 (1–137) co-immunoprecipitated with FBP1 all exons, D3 co-immunoprecipitated with FBP1 exon 2, and D6 co-immunoprecipitated with FBP1 exon 3, 4 (Fig. 4H).

To determine whether FBP1 affected the binding of STAT3 to the promoter of STAT3-mediated target genes, we performed chromatin immunoprecipitation (ChIP) assay to test the binding of STAT3 to the promoter of MMP3, Bcl-2, and HIF-1α, which had been identified as STAT3-mediated genes, in A2780 cells introduced with FBP1 cDNA or control vector. As shown in Fig. 4I, we found that overexpression of FBP1 obviously weakened the binding of STAT3 to the promoter of MMP3, Bcl-2 and HIF-1α, indicated that FBP1 exerted its inhibitory function on cell proliferation, metastasis, chemoresistance by blocking the binding of STAT3 to the promoter of STAT3-mediated genes.

To determine whether the metabolic enzymatic activity of FBP1 is required to inhibit STAT3, we expressed a previously described, catalytically inactive FBP1 G260R mutant [13] in A2780 and SKOV3 cells (Supplementary Fig. 9A, B). By Co-immunoprecipitation, we confirmed that FBP1 G260R also bound with STAT3 (Supplementary Fig. 9C). Moreover, FBP1 G260R overexpression significantly reduced the accumulation of STAT3 protein in the nucleus, whereas had no impact on cytoplasmic levels of STAT3 (Supplementary Fig. 9D). Further co-immunoprecipitation (Supplementary Fig. 9E), FRET-FLIM (Supplementary Fig. 9E, F), and immunofluorescence assay (Supplementary Fig. 9G) demonstrated that this direct interaction can prevent STAT3 nuclear translocation. Functionally, FBP1 G260R inhibited cell growth to a comparable level of wild-type FBP1 at 10 mM glucose (Supplementary Fig. 10A). FBP1 G260R also influenced colony formation (Supplementary Fig. 10B), metastasis (Supplementary Fig. 10C), glucose metabolism (Supplementary Fig. 10D–E), apoptosis (Supplementary Fig. 10F–G), and STAT3 target gene expression (Supplementary Fig. 10H) to the same extent of wild-type FBP1 in A2780 and SKOV3 cells. These results suggest that FBP1 interferes with STAT3 function through a metabolic catalytic activity-independent mechanism.

Next, we determined whether the introduction of STAT3 cDNA could reverse the effect of FBP1 in ovarian cancer cells. We transiently overexpressed STAT3 cDNA in FBP1-overexpressing A2780 and SKOV3 cells. Induction of STAT3 cDNA strongly suppressed the inhibition of cell migration (Supplementary Fig. 11A, B), cell proliferation (Supplementary Fig. 11C, D), and glycolysis (Supplementary Fig. 11E, F) mediated by FBP1-overexpressing. Consistently, western blotting results also confirmed that the FBP1-mediated decrease in Bcl-2, MMP3, GLUT4, and LDHA protein levels were rescued by overexpression of STAT3, however, the increase in BAX and E-cadherin was suppressed by overexpression of STAT3 (Supplementary Fig. 11G). We further found that STAT3 significantly attenuated FBP1-overexpressing induced cisplatin sensitivity in both A2780 and A2780·cis cells (Supplementary Fig. 11H), which was also confirmed by the western blotting results (Supplementary Fig. 11I). In vivo studies also confirmed the role of STAT3 in the effect of FBP1 in ovarian cancer cells with or without cisplatin treatment (Supplementary Fig. 12).

### FBP1 is directly regulated by C-MYC in ovarian cancer

**Fig. 5:**
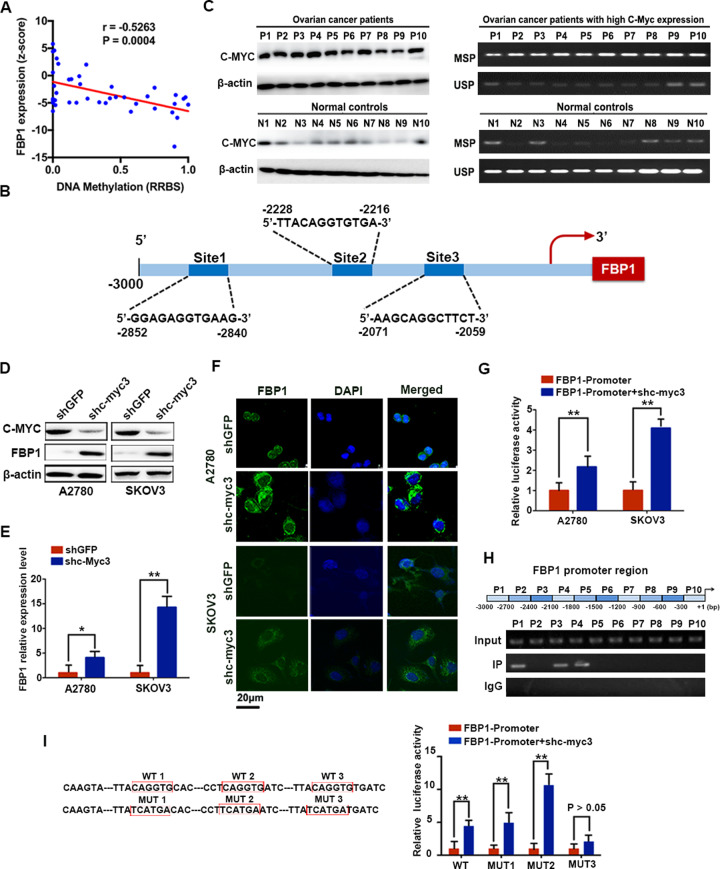
FBP1 was regulated directly by C-MYC in ovarian cancer cells. **A** The correlation between FBP1 mRNA expression level and the degree of methylation. **B** Enrichment of C-MYC at the promoter of FBP1, figure was download from UCSC Genome Bioinformatics Site (http://genome.ucsc.edu/). **C** Representative images of methylation-specific PCR results from ovarian cancer patients and healthy women. **D** Western blotting analysis of FBP1 protein expression in cells with the silencing of c-myc. **E** RT-qPCR analysis of FBP1 mRNA expression in cells with the silencing of c-myc. **F** Representative immunofluorescence staining images (×1000) showing that C-MYC inhibited the expression of FBP1 in the cytoplasm (green). Blue dye (DAPI) indicates the nucleus. **G** Luciferase reporter assay in A2780 and SKOV3 cell lines with c-myc downregulated. **H** The results of ChIP analysis showed that C-MYC can bind to the FBP1 promoter region. **I** Luciferase reporter assay of C-MYC mutant sites in the promoter region of FBP1. *P < 0.05, **P < 0.01.

Our results suggested that FBP1 was downregulated in ovarian cancer and that this could be related to tumor progression; therefore, next we aim to investigate the mechanisms of FBP1 downregulation in this type of cancer. The downregulation of FBP1 expression due to promoter DNA methylation has been observed in multiple human malignancies [10, 16, 17]. Therefore, we compared the FBP1 expression level and FBP1 promoter DNA methylation levels across 47 ovarian cancer cell lines from the Cancer Cell Line Encyclopedia (CCLE) [20]. We found that 63.08% (41/65) of ovarian cancer cell lines from the CCLE database harbored FBP1 promoter DNA methylation and the degree of methylation was negatively correlated with the FBP1 mRNA expression level **(**Fig. 5A).

To investigate which transcription factors were involved in DNA methylation-mediated regulation of FBP1 expression, we investigated transcription factor binding at the FBP1 promoter. We analyzed the ENCODE TFBS ChIP sequencing (ChIP-seq) data and the genomic locus information for FBP1 from UCSC (http://genome.ucsc.edu), and we found that C-MYC was highly enriched at the FBP1 promoter (Fig. 5B). Interestingly, C-MYC has been reported to inhibit the expression of its target genes by inducing promoter DNA methylation in human breast cancer [21]. Indeed, methylation-specific PCR analysis of ovarian tissue samples showed that compared to healthy ovarian tissue (*n* = 10), ovarian cancer patients (*n* = 10) with high C-MYC expression levels exhibited high DNA methylation of FBP1 (Fig. 5C). We also found a negative relationship between endogenous FBP1 and C-MYC protein levels in four ovarian cancer cell lines (Supplementary Fig. 13A). Accordingly, FBP1 was upregulated when C-MYC was knocked down in A2780 and SKOV3 cells (Supplementary Fig. 13B, Fig. 5D–E), which was validated by the result of immunofluorescence experiments that confirmed an increase in FBP1 protein levels in the cytoplasm is associated with C-MYC knockdown (Fig. 5F). These results indicate that FBP1 is regulated by C-MYC and promoter DNA methylation in ovarian cancer.

Next, we constructed a FBP1 promoter luciferase reporter plasmid and performed a luciferase reporter assay to confirm the mechanistic link between FBP1 and C-MYC. Firstly, we transfected FBP1 promoter plasmids into A2780 and SKOV3 cell lines with transient shC-MYC plasmid. Compared with the control groups, knockdown of c-myc significantly increased luciferase expression (Fig. 5G). To confirm the exact region within the FBP1 promoter that C-MYC binds, we performed ChIP in A2780 cell lines. These experiments identified three C-MYC binding sites existed at approximately 1800–2400 bp and 2700–3000 bp upstream of the open reading frame (ORF) of FBP1 (Fig. 5H). We individually mutated each of these binding sites and repeated the luciferase assay, and showed that mutation of the third binding site alone abrogated luciferase activity, suggesting that this was a C-MYC binding site (Fig. 5I). Together, the above data indicate that FBP1 is a vital target gene of C-MYC and C-MYC may suppress FBP1 promoter activity by DNA methylation.

### Abrogation of FBP1 expression attenuates tumor-suppressive properties mediated by C-MYC silencing

To further demonstrate that FBP1 is a critical target gene of C-MYC, we performed the rescue experiment by knocking down the expression of C-MYC and FBP1 with shRNA and observed the impact on cell proliferation, invasion, migration, cisplatin-induced apoptosis and glycolysis. In both cell lines, FBP1 depletion partially reversed the reduction of cell growth and migration caused by the knockdown of C-MYC (Supplementary Fig. 13C, D). In addition, FBP1 knockdown significantly reverted C-MYC knockdown-mediated decrease in glucose consumption and lactose production (Supplementary Fig. 13E, F), as well as the C-MYC knockdown-mediated sensitization to cell death induced by cisplatin (Supplementary Fig. 13G). By western blotting, we also observed that FBP1 and C-MYC depletion decreased expression of the pro-apoptotic protein BAX while increased the anti-apoptotic protein Bcl-2 and EMT-related protein MMP3 in both A2780 and A2780·cis cell lines when they were pretreated with cisplatin (Supplementary Fig. 13H), and FBP1 rescued the expression of C-MYC-mediated proteins involving in cell proliferation, invasion, migration, apoptosis, and glycolysis (Supplementary Fig. 13I). Taken together, these findings demonstrate that may promote tumor progression and cisplatin resistance by suppressing FBP1 expression.

### Expression of C-MYC, STAT3, and p-STAT3 is related to poor survival in ovarian cancer patients

**Fig. 6:**
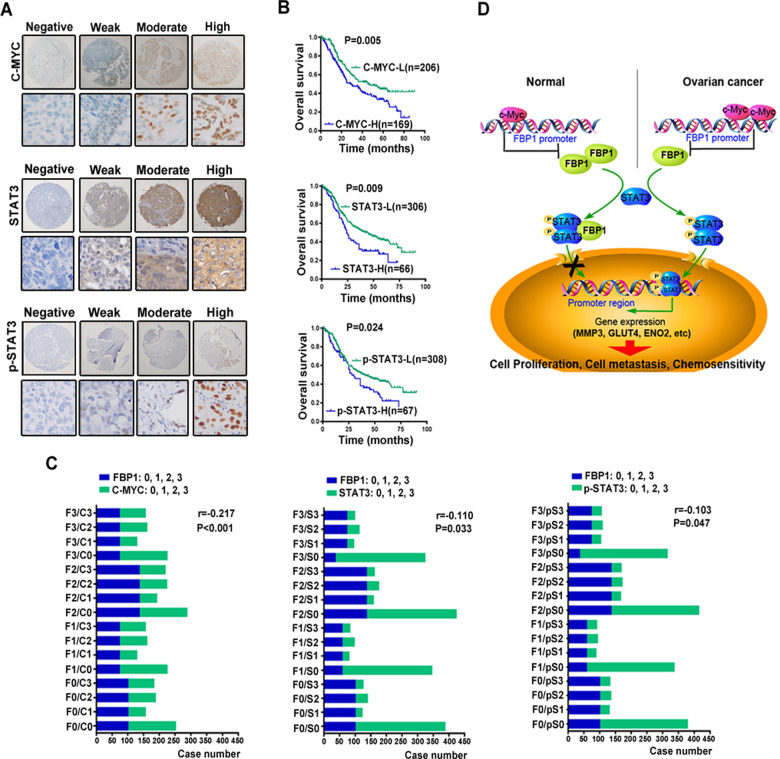
Immunohistochemical staining of C-MYC, STAT3, and p-STAT3 in ovarian cancer. **A** Representative images of biopsies containing negative, weak, moderate, and strong expression of C-MYC, STAT3, and p-STAT3 (all 400×). **B** Survival analysis of patients by Kaplan–Meier plots and log-rank tests. Patients were categorized as having high or low expression of C-MYC, STAT3, and p-STAT3 on the basis of IHC staining scores. H high; L low. Only IHC scores ≥3 were considered high. **C** Correlation of IHC scores between FBP1 and C-MYC, STAT3, and p-STAT3. **D** Schematic model showing the role of C-MYC-FBP1-STAT3 signaling axis in the regulation of cell proliferation, metastasis, and chemosensitivity.

To ascertain the clinical significance of C-MYC, STAT3, and p-STAT3 in ovarian cancer, we assessed their expression in an ovarian tissue microarray (*n* = 375). C-MYC was expressed at high levels in 45.1% (169/375) of ovarian cancer tissues, 66 (14.9%) patients showed “high” expression of STAT3, and 67 (15.1%) patients showed “high” expression of p-STAT3 (Fig. 6A, Supplementary Tables 1 and 2). High expression of C-MYC (log-rank, *P* = 0.005), STAT3 (log-rank, *P* = 0.009) and p-STAT3 (log-rank, *P* = 0.024) were all correlated with poor OS (Fig. 6B). The correlation of FBP1, C-MYC, STAT3, and p-STAT3 with clinicopathological characteristics in ovarian cancer patients were showed in Supplementary Table 2. In agreement with our earlier observations, C-MYC expression was inversely correlated with FBP1 expression (*r* = −0.217, *P* < 0.001), in addition, STAT3 (*r* = −0.110, *P* = 0.033) and p-STAT3 (*r* = −0.103, *P* = 0.047) expression levels were also inversely correlated with FBP1 expression (Fig. 6C). Finally, we identify a C-MYC-FBP1-STAT3 signaling axis in ovarian tumorigenesis (Fig. 6D).

## Discussion

Here, we identified an anticancer role of FBP1 and a C-MYC-FBP1-STAT3 axis in ovarian tumorigenesis, which could yield a potential future molecular marker for the chemosensitivity and prognosis of epithelial ovarian carcinoma.

First, we observed that downregulation of FBP1 was prevalent in ovarian tumor tissues and was associated with poor prognosis. We then found that enhanced FBP1 efficaciously reduced the malignancy of cancer cells, whereas FBP1 depletion did the opposite. FBP1 was originally identified as the rate-limiting enzyme in gluconeogenesis and loss of FBP1-mediated metabolic reprogramming that resulted in malignant behavior in cancer cells [10]. In agreement with previous studies [22, 23], our data showed that FBP1 strongly reduced the glucose metabolism. Also its direct role in tumorigenesis and tumor progression are gradually identified [13]. In consistent with one recent study [24], we found that STAT3 is a potential target of FBP1 in cancer cells. Interestingly, in addition to directly binding to the transactivation domain of STAT3, our further research found that FBP1 also bound to the domain containing the nuclear translocation signal of STAT3, thus detaining STAT3 in the cytoplasm. This finding reveals the mechanism underlying the suppressive effect of FBP1 on cell proliferation, apoptosis, migration, and invasion in cancer cells.

Second, we discovered that C-MYC interacted directly with the FBP1 promoter and regulated the expression of FBP1 mRNA. C-MYC is a well-known oncogene that is frequently upregulated in different malignancies and plays a pivotal role in promoting cell growth and proliferation [25, 26]. It has been reported before that C-MYC might be an indirect downstream molecule [27–29]. However, as a transcription factor, C-MYC transcriptionally regulates target gene involved in glycolytic flux [30, 31]. Previous studies have shown that hypermethylation of the FBP1 promoter region is the main cause of loss of FBP1 in various cancers [9, 10, 15–17]. Our results suggested that C-MYC directly bound to the promoter region of FBP1 and promoted its methylation, reinforcing the importance of promoter methylation in abrogating FBP1 expression. Furthermore, reducing FBP1 expression in ovarian cancer cells reversed the inhibition of cell progression induced by C-MYC knockdown.

Thus, this novel C-MYC-FBP1 signaling axis critically contributed to the Warburg effect in ovarian cancer cells and, as a result, to the development and progression of ovarian cancer.

Third, we found that overexpression of FBP1 conferred sensitivity to cisplatin. Platinum resistance has always been a serious challenge in the treatment of ovarian cancer patients. The persistent accumulation of irreparable double-strand breaks (DSBs) was recognized as the main mechanism of cisplatin-induced cancer cell death [32]. Therefore, meformin was proven to enhance the effect of cisplatin in ovarian cancer due to its impact on DNA damage [33, 34]. In addition, cisplatin can also exert an inhibitory effect on glycolysis in cancer cells in recent studies. Currently, combined treatment modalities that target glycolytic pathways hold promise for the treatment of chemoresistant cancer cells [35]. Inhibition of glycolysis enhances drug-induced apoptosis in ovarian cancer, lung cancer, and leukemia [36–38]. Therefore, inhibition of glycolysis by targeting FBP1 may play a vital role in restoring cisplatin sensitivity. To our knowledge, this is the first time that FBP1 has been linked to chemosensitivity in ovarian cancer.

In sum, our data provided mechanistic insight into the role of FBP1 and C-MYC-FBP1-STAT3 axis in the tumorigenesis and progression of ovarian cancer. Therefore, FBP1 may warrant consideration as a predictive biomarker for the individual response of ovarian cancer patients to chemotherapy in prospective clinical studies.

## Materials and method

### Patients and tissue samples

Tissue microarrays were made using high-grade serous adenocarcinoma samples from 375 formalin-fixed paraffin-embedded lesions of the ovarian cancer patients with FIGO (International Federation of Gynecology and Obstetrics, 2014) stages I–IV and 23 cervical cancer patients with normal ovary between March 2008 and March 2012 available at the tissue bank of Fudan University Shanghai Cancer Center (FUSCC). Each patient’s follow-up was initiated at the beginning of chemotherapy. All the 375 patients underwent cytoreduction surgery combined with postoperative platinum and taxane therapy, and the other patients just underwent cytoreduction surgery. Disease-free time (DFS) was calculated from the end of chemotherapy to the date of clinically proven progression. OS was defined as the length of time from the date of diagnosis to the date of cancer-related death or the last visit. DFS > 6 months was defined as sensitivity to the last platinum-based chemotherapy, and DFS ≤ 6 months was defined as resistant to the last platinum-based chemotherapy. All studies involving human participants were approved by Ethics Committee at FUSCC. A written informed consent was approved from all recruited individuals, and each clinical investigation was conducted according to the principles expressed in the Declaration of Helsinki consent.

### Cell lines and culture

The established human ovarian cancer cell lines were obtained from the Cell Bank of the Chinese Academy of Science. All cells were maintained in Dulbecco’s modified Eagle’s medium (DMEM, HyClone, Thermo Scientific, USA) supplemented with 10% fetal bovine serum (Gibco, Life technologies, USA), 100 U/ml penicillin (Biowest, Nuaillé, France), and 100 U/ml streptomycin (Biowest, Nuaillé, France) and incubated at 37 °C in a humidified atmosphere with 7% CO_2_.

### Chromatin immunoprecipitation (ChIP) assay

ChIP assays were performed using Pierce Agarose ChIP Kit (Thermo, #27177). Briefly, A2780 were crosslinked by 1% formaldehyde for 10 min at 37 °C. The cross-linking reaction was quenched by glycine and cells were lysed in SDS buffer containing protease inhibitor cocktail. Cell lysates were sonicated to shear chromatin DNA into fragments with 200–1000 base pairs in size and then subjected to immunoprecipitation with 4 μl IgG (Cell Signaling Technology), 7μl C-MYC (ab32, mouse monoclonal antibody, Abcam) or STAT3 (#9139, mouse monoclonal antibody, Cell Signaling Technology) or 2 μl Polymerase II (Imgenex) antibodies. After washing with a series of low and high salt concentration washing buffers, immunoprecipitated DNA fragments were de-crosslinked at 77 °C in high salt condition, purified using QIAquick PCR purification kit (Qiagen), and then analyzed by qRT-PCR.

Using the GAPDH promoter primers (Supplementary Table 3) confirmed the effectiveness of conventional PCR chip results. The correct chip results should be that only the input and RNApolII samples will have positive results, which could be shown as a 300 bp band PCR, and the other three groups (IgG, C-MYC, and STAT group 3) appeared no band. In the ORF region of human FBP1 gene, which located within upstream 3000 bp long of the target gene, a pair of primers was designed by using of Primer7.0 every 300 bp or so.

### Statistical analysis

The data in this study were calculated using Graph Pad Prism and reported as mean ± SD. Clinico-pathologic characteristics analysis was performed using SPSS 23.0 (SPSS Inc., Chicago, IL). Comparisons between controls and treated groups were determined by paired *t* test or one-way ANOVA followed by Tukey’s multiple comparison tests. The relationship between FBP1 and C-MYC, STAT3, and p-STAT3 was conducted using Spearman Correlation Coefficient. The association between FBP1, C-MYC, STAT3, and p-STAT3 expression and clinicopathological characteristics was evaluated using the *χ*^2^ test. The Kaplan–Meier method with log-rank analysis was used to obtain estimates of DFS and OS. Variables with a value of *p* < 0.05 in univariate analysis were included in subsequent multivariate analysis on the basis of the Cox proportional hazards model. A probability value of less than 0.05 was considered statistically significantly different.

## Supplementary information

Figure S1

Figure S2

Figure S3

Figure S4

Figure S5

Figure S6

Figure S7

Figure S8

Figure S9

Figure S10

Figure S11

Figure S12

Figure S13

Table S1

Table S2

Table S3

Supplementary Figure Legends

Supplemental Material and Methods
