# Supplementary figures and images for: FBP1 regulates proliferation, metastasis, and chemoresistance by participating in C-MYC/STAT3 signaling axis in ovarian cancer
Source: Oncogene. 2021 Aug 6;40(40):5938–49. doi: 10.1038/s41388-021-01957-5 (PMC8497274; doi:10.1038/s41388-021-01957-5)

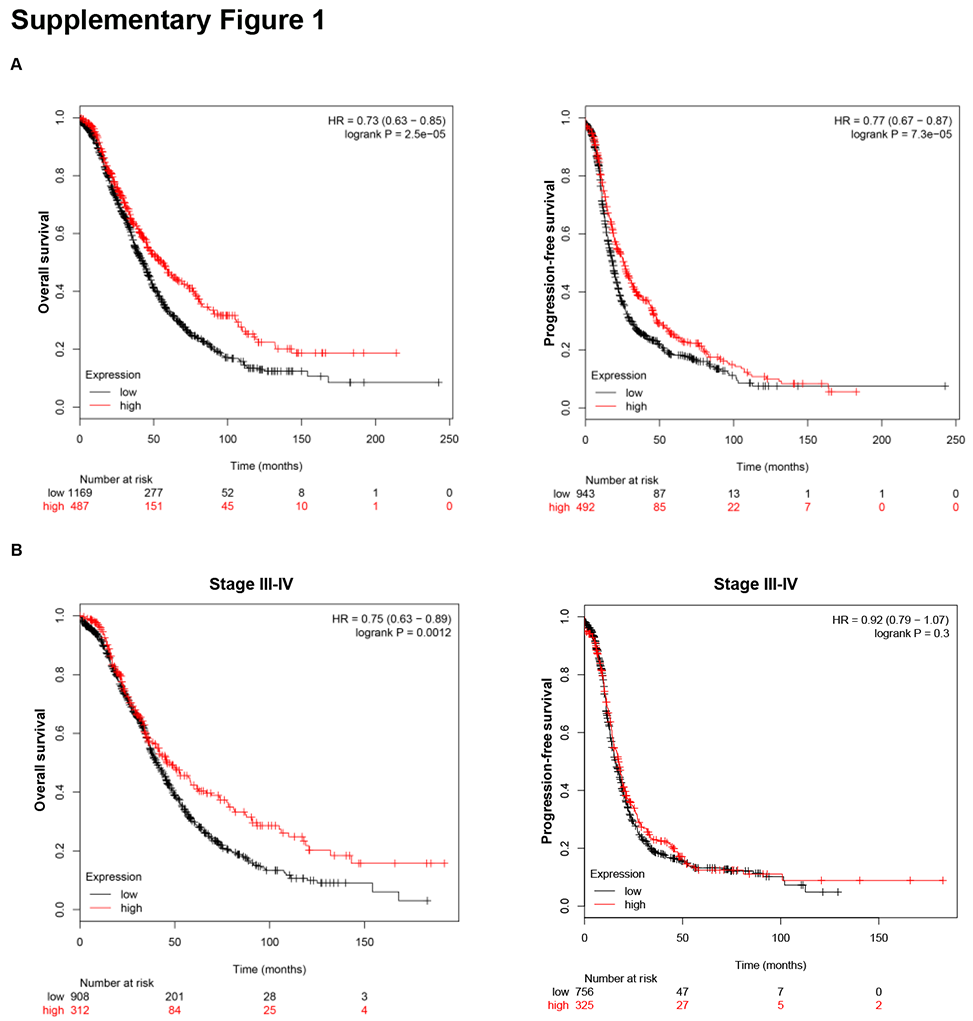

Supplement: Supplementary file 1 — Figure S1 [file 41388_2021_1957_MOESM1_ESM.tif]

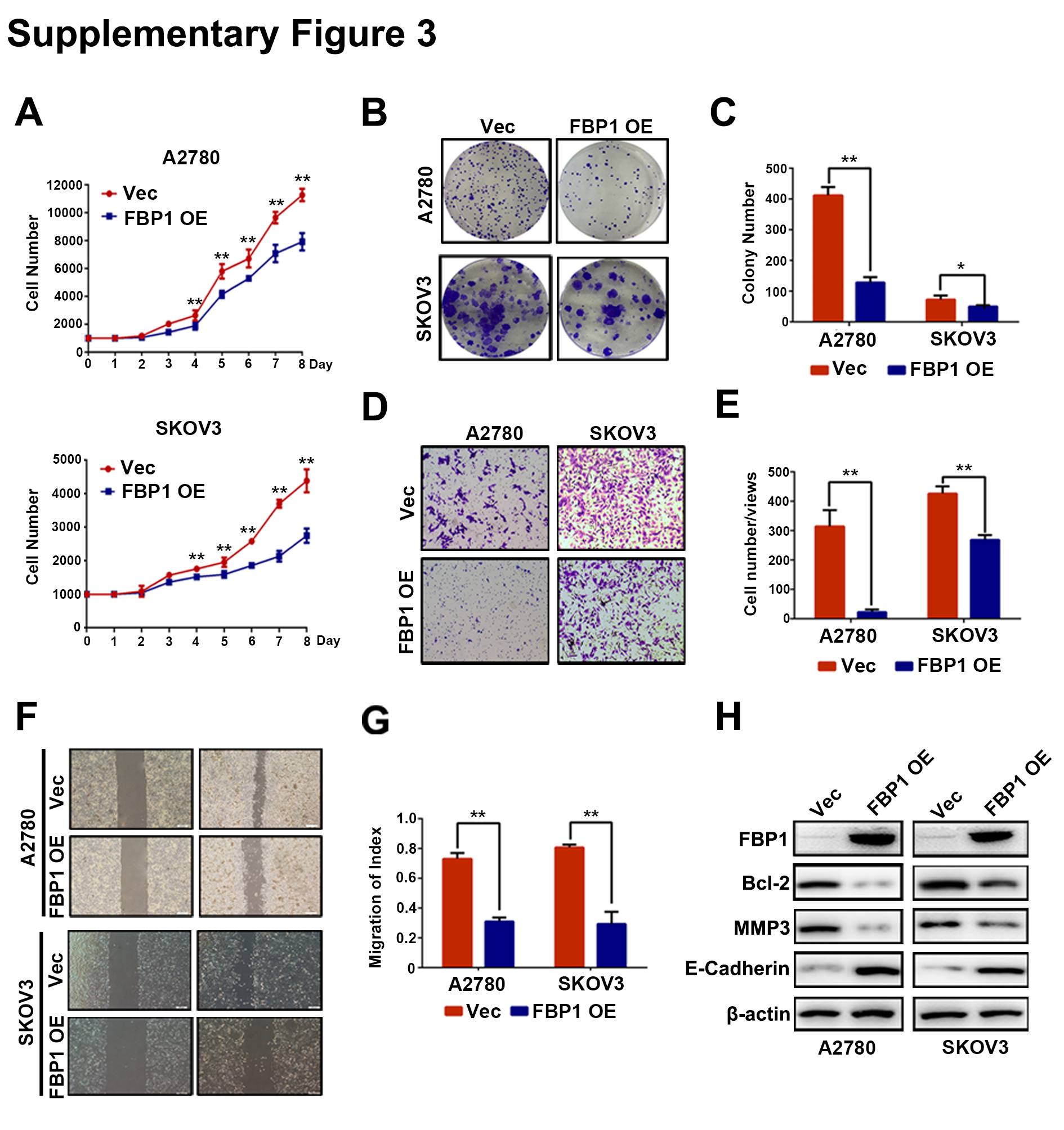

Supplement: Supplementary file 3 — Figure S3 [file 41388_2021_1957_MOESM3_ESM.tif]

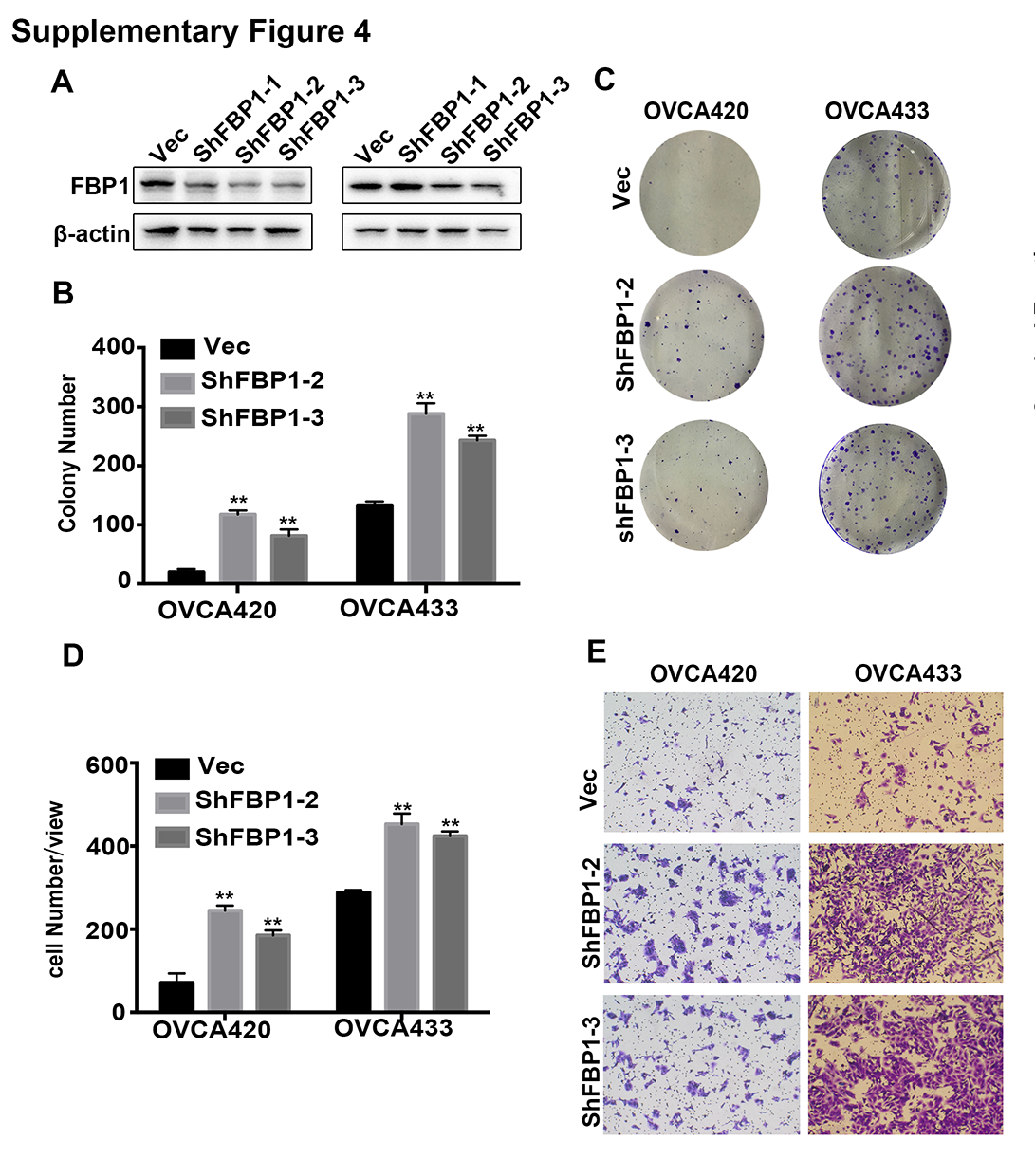

Supplement: Supplementary file 4 — Figure S4 [file 41388_2021_1957_MOESM4_ESM.tif]

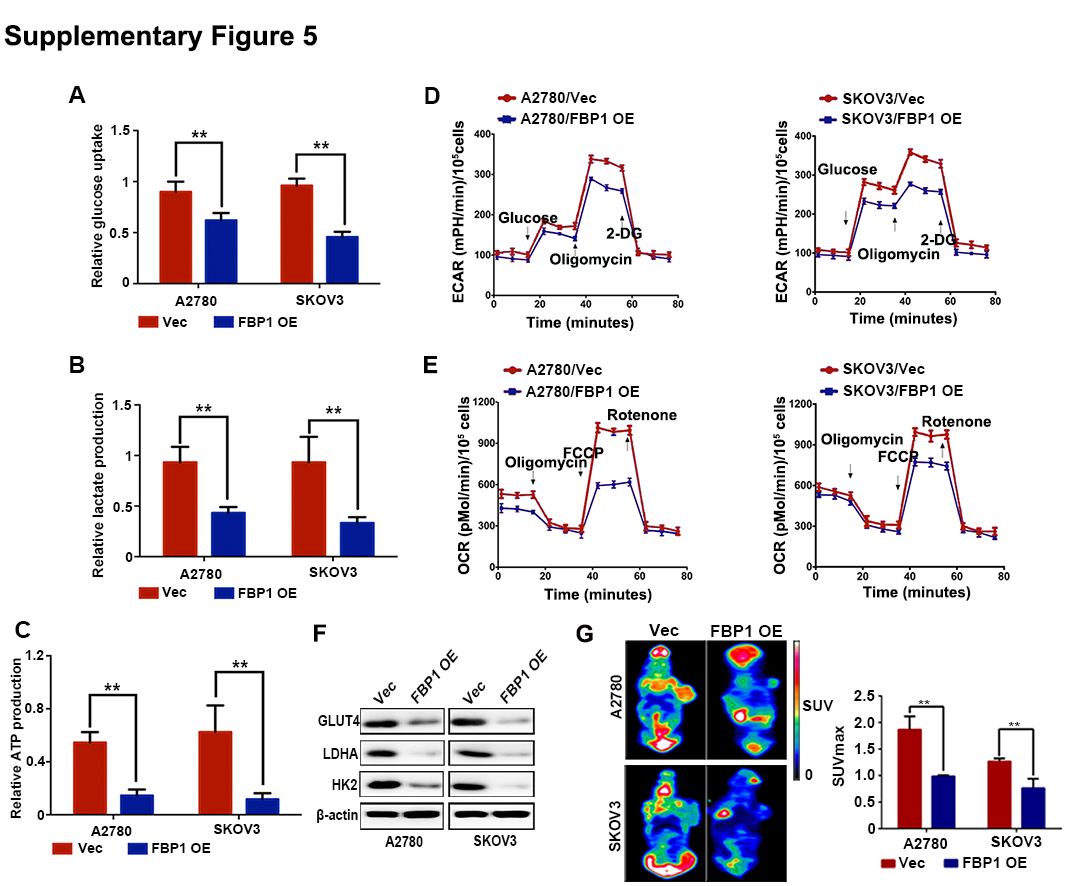

Supplement: Supplementary file 5 — Figure S5 [file 41388_2021_1957_MOESM5_ESM.tif]

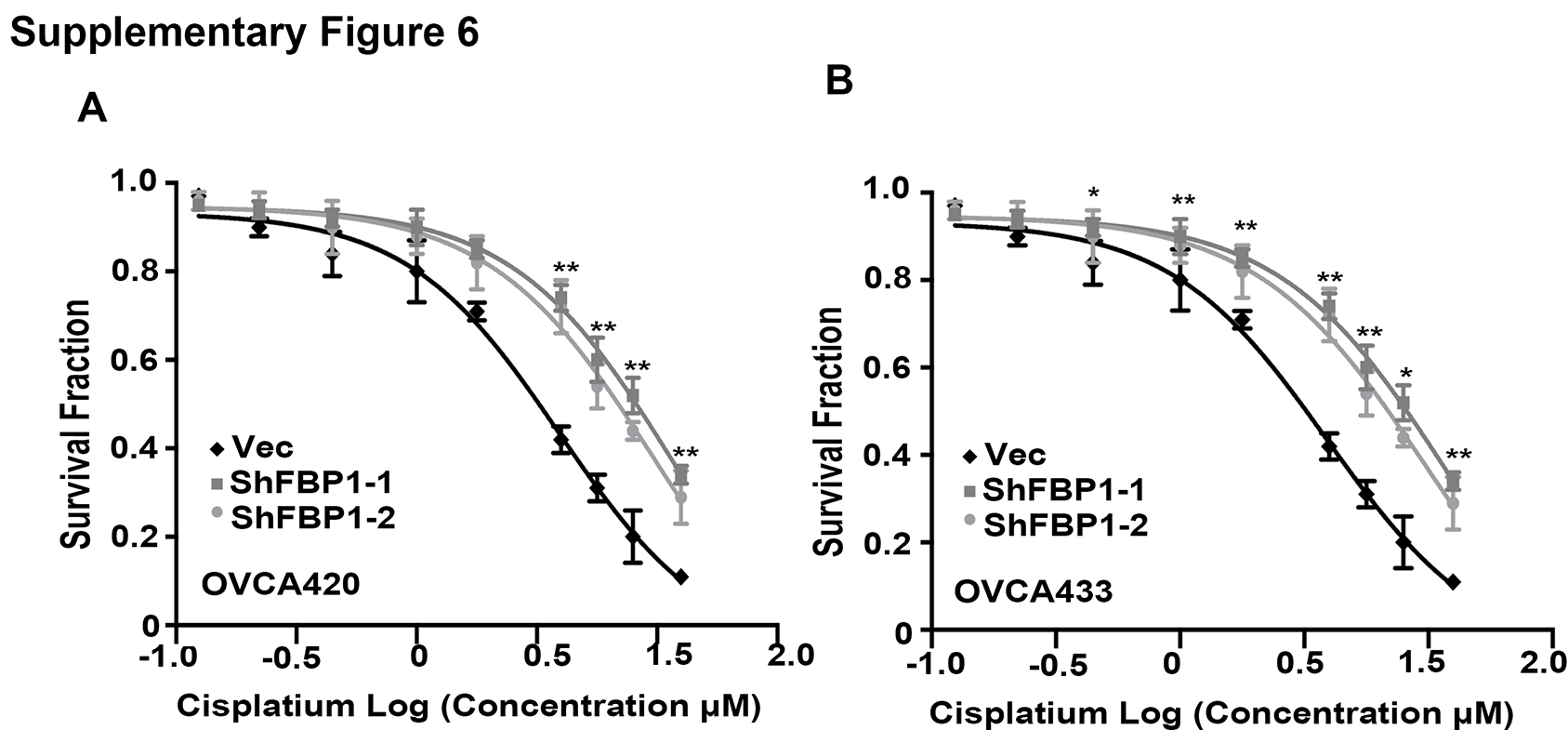

Supplement: Supplementary file 6 — Figure S6 [file 41388_2021_1957_MOESM6_ESM.tif]

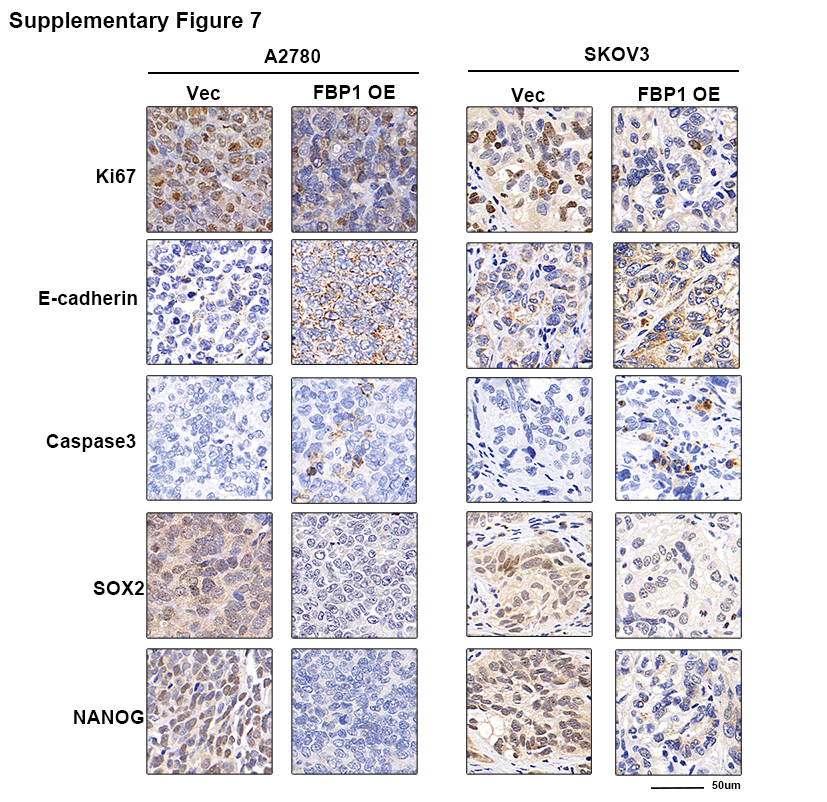

Supplement: Supplementary file 7 — Figure S7 [file 41388_2021_1957_MOESM7_ESM.tif]

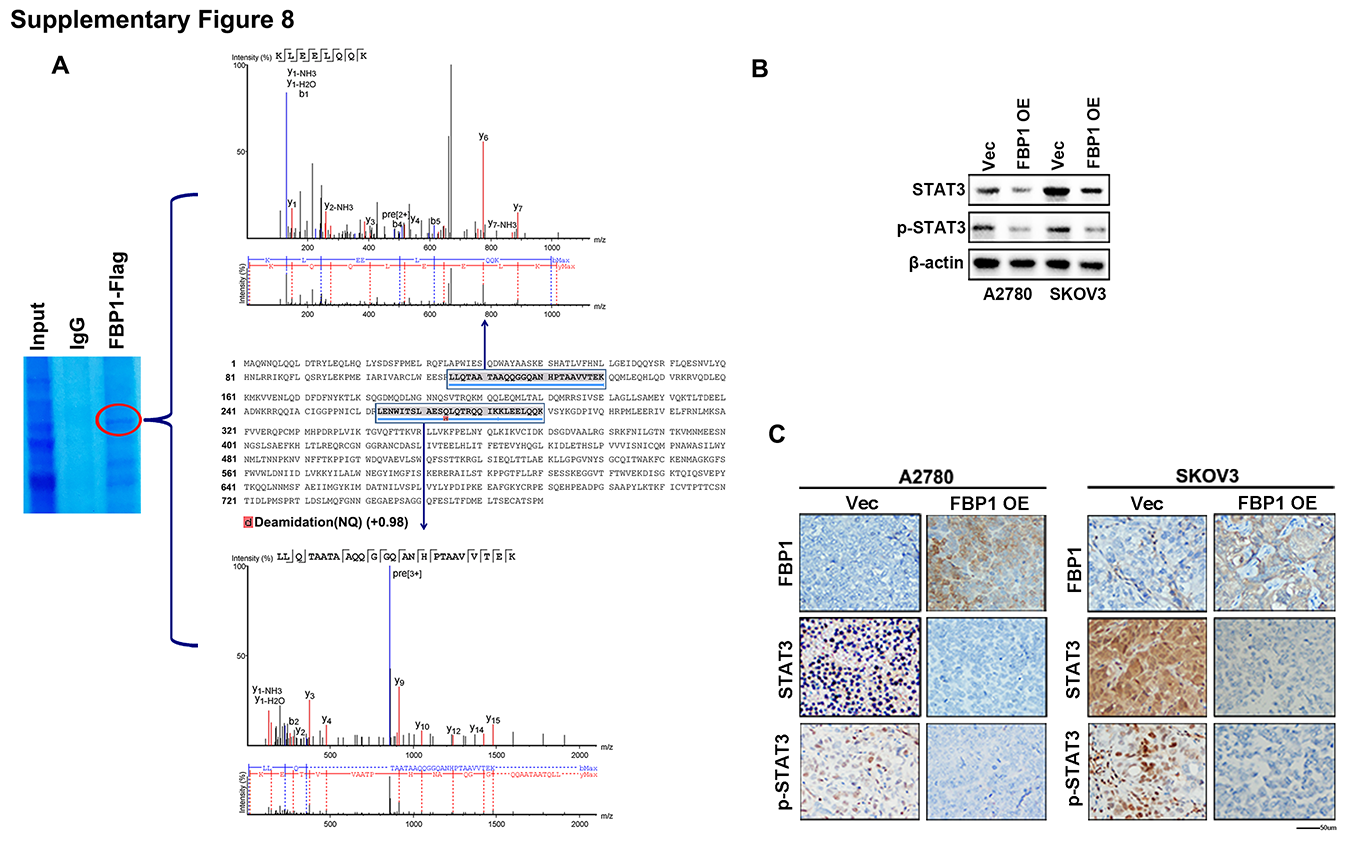

Supplement: Supplementary file 8 — Figure S8 [file 41388_2021_1957_MOESM8_ESM.tif]

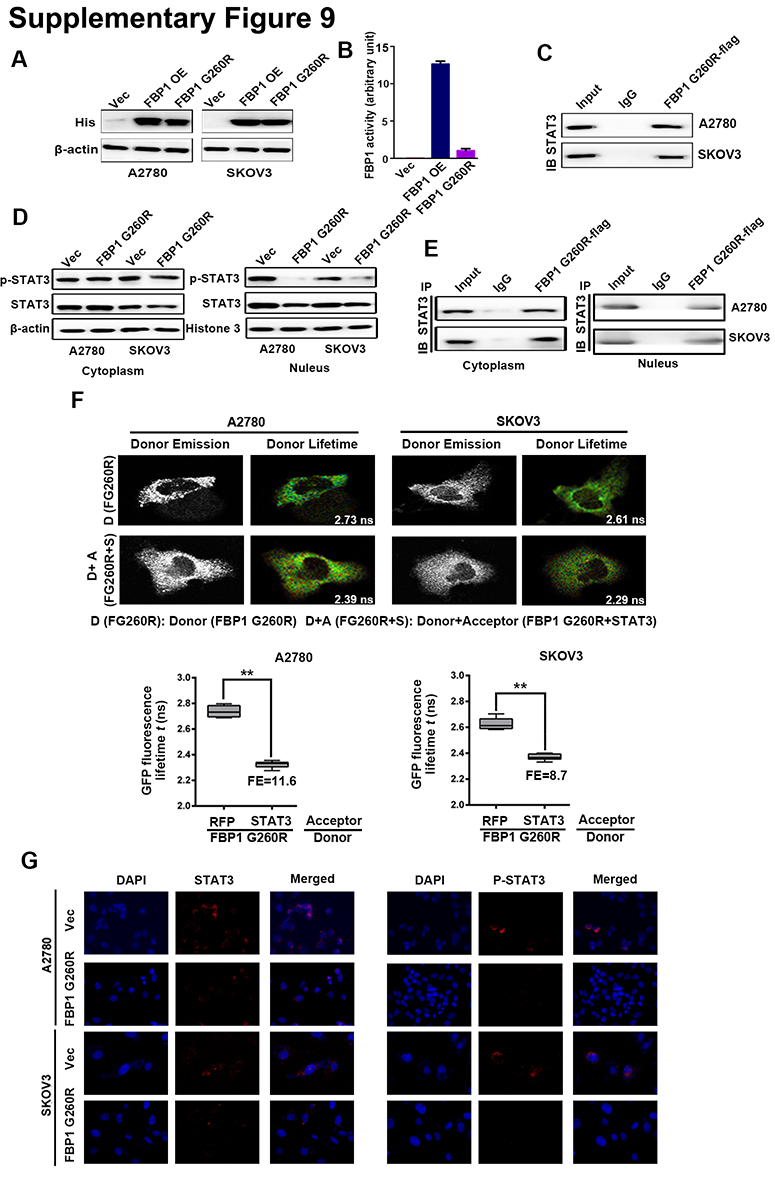

Supplement: Supplementary file 9 — Figure S9 [file 41388_2021_1957_MOESM9_ESM.tif]

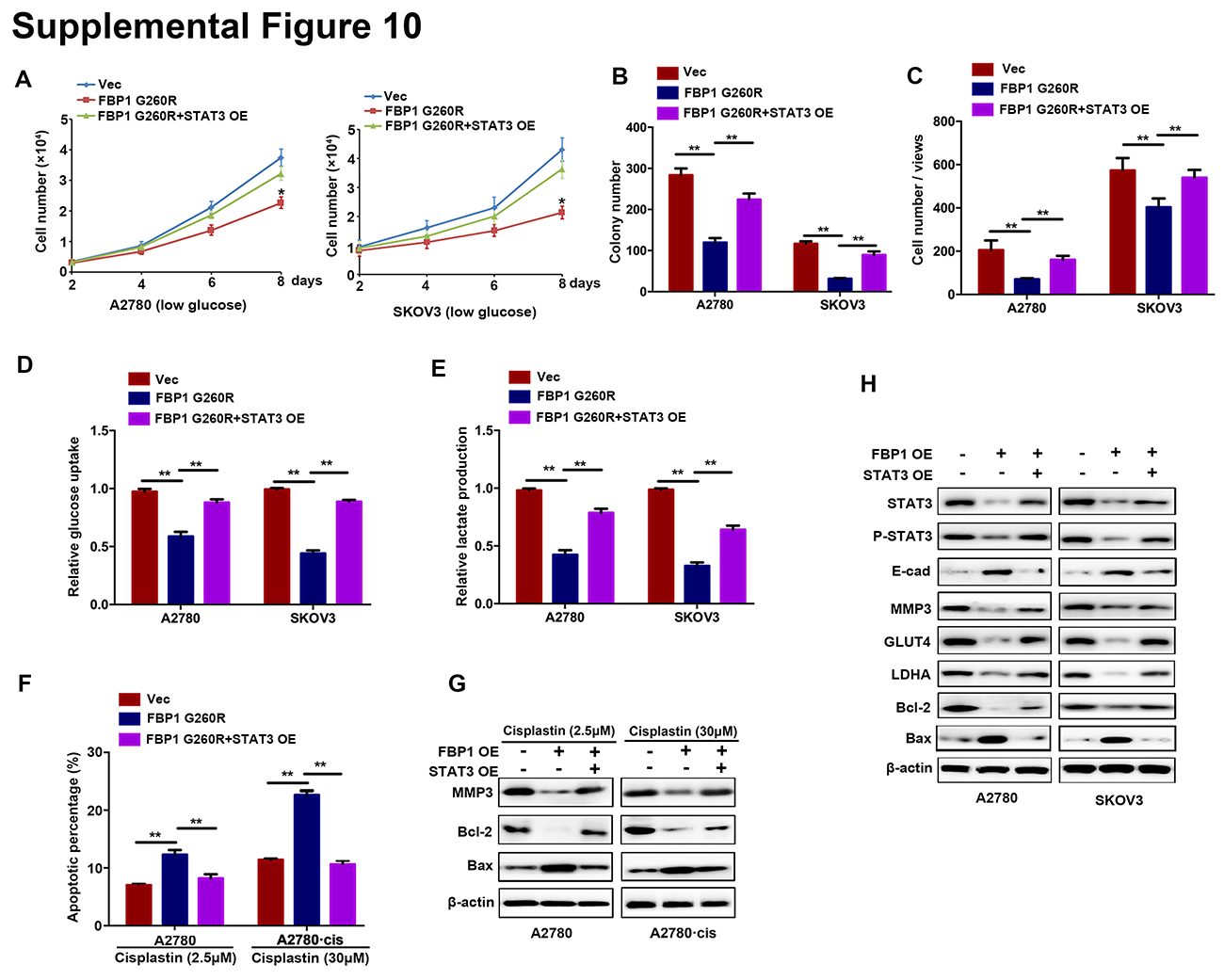

Supplement: Supplementary file 10 — Figure S10 [file 41388_2021_1957_MOESM10_ESM.tif]

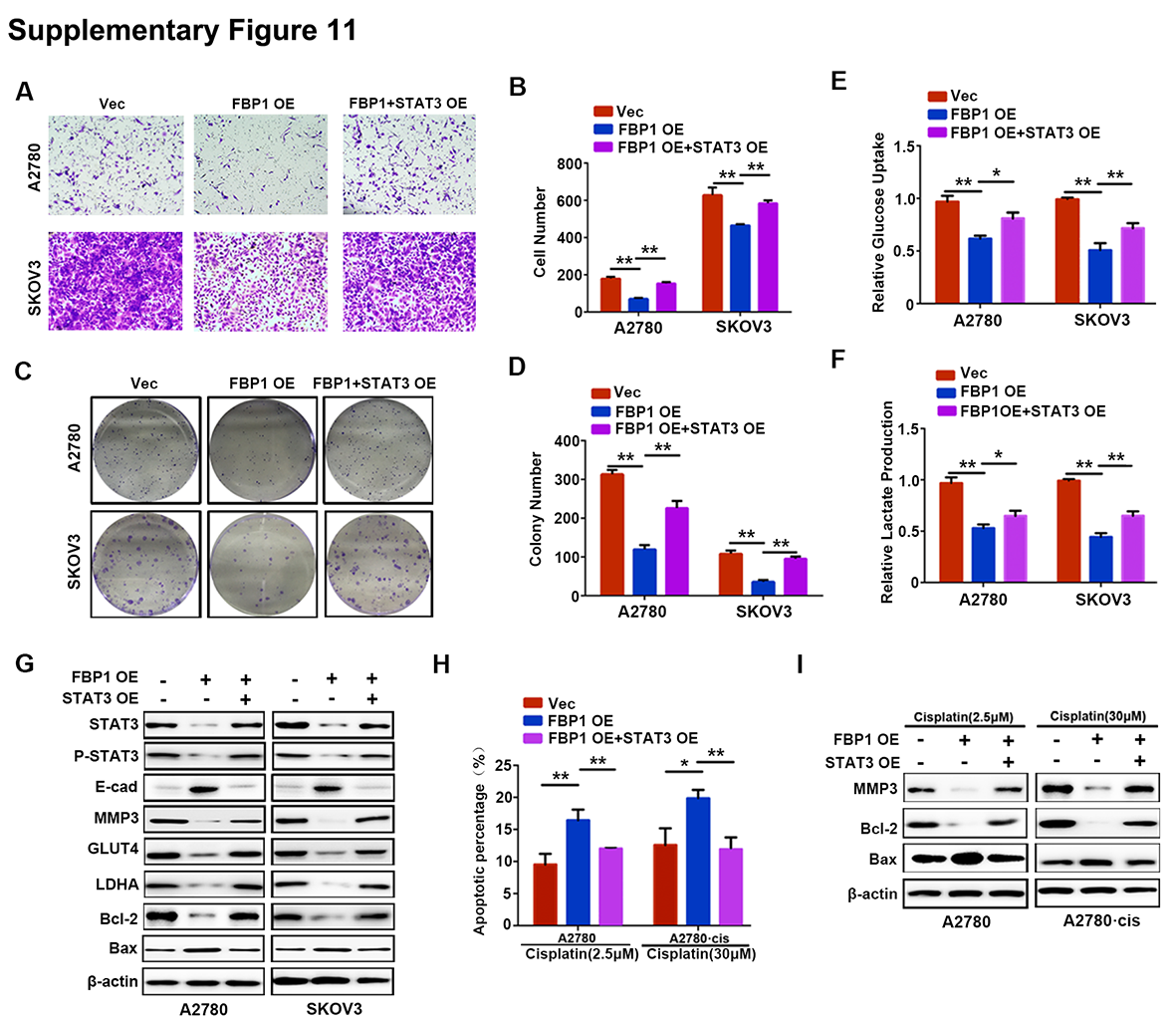

Supplement: Supplementary file 11 — Figure S11 [file 41388_2021_1957_MOESM11_ESM.tif]

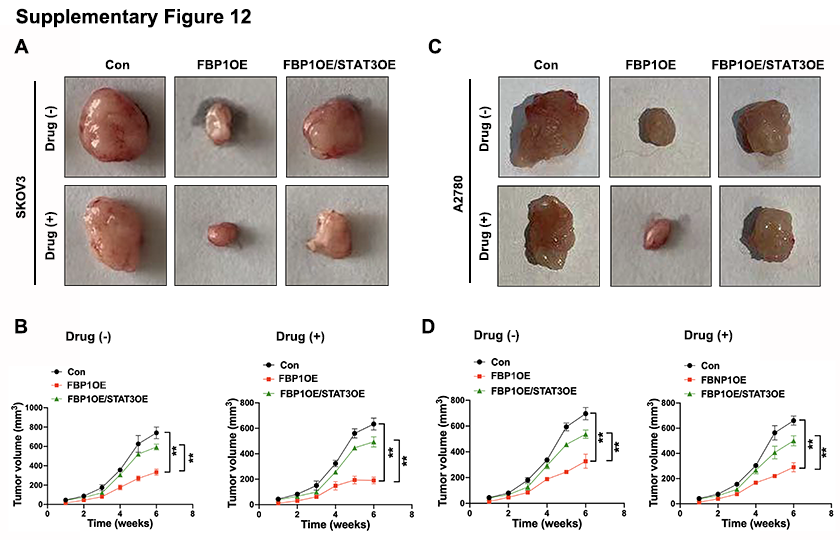

Supplement: Supplementary file 12 — Figure S12 [file 41388_2021_1957_MOESM12_ESM.tif]

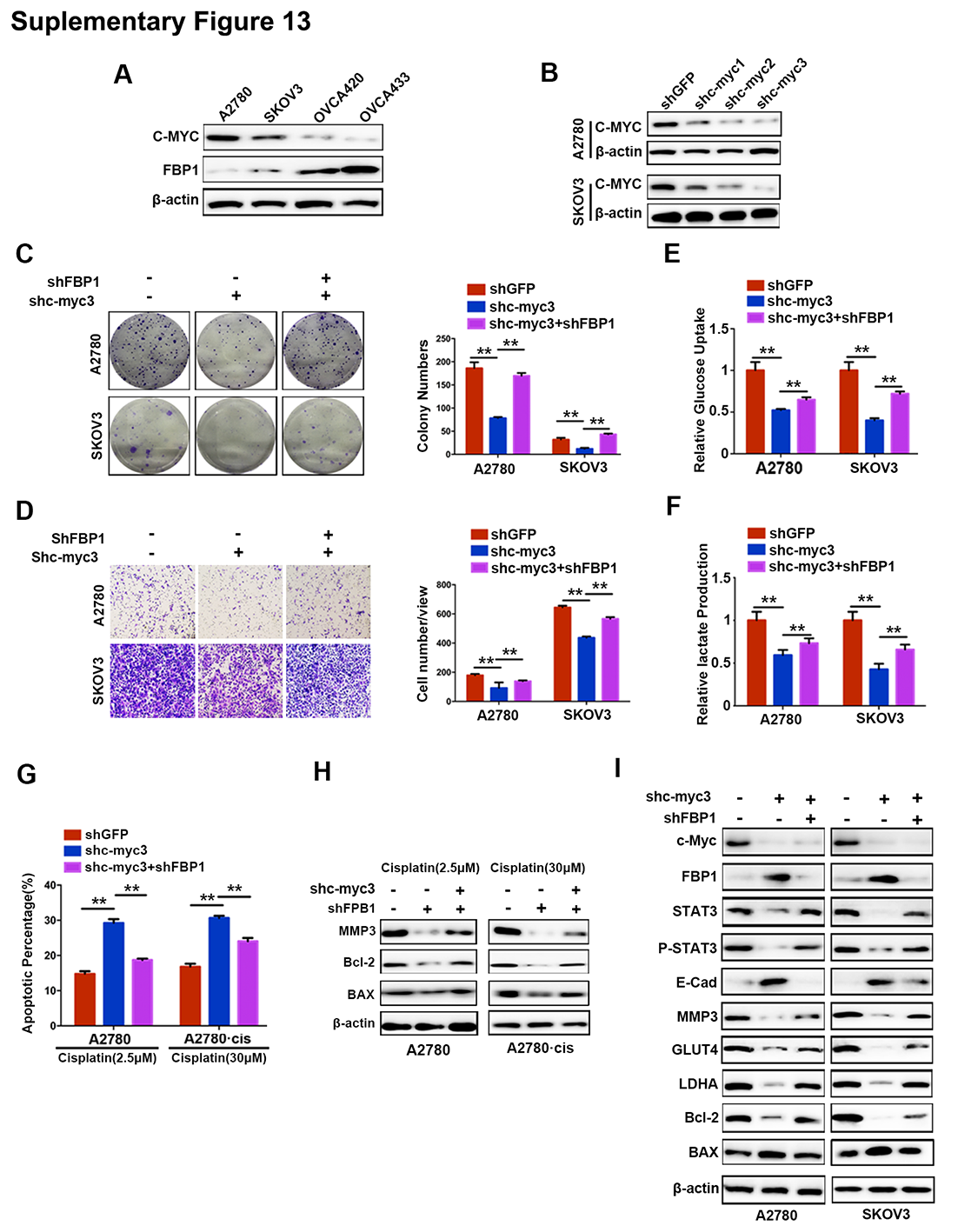

Supplement: Supplementary file 13 — Figure S13 [file 41388_2021_1957_MOESM13_ESM.tif]
