# Supplementary material for: FBP1 regulates proliferation, metastasis, and chemoresistance by participating in C-MYC/STAT3 signaling axis in ovarian cancer
Source: Oncogene. 2021 Aug 6;40(40):5938–49. doi: 10.1038/s41388-021-01957-5 (PMC8497274; doi:10.1038/s41388-021-01957-5)
Supplement: Supplementary file 14 — Table S1 [file 41388_2021_1957_MOESM14_ESM.docx]

**Supplementary Table 1. The association between clinicopathological characteristics and overall survival in ovarian cancer patients**

| Prognostic factors | Patients N (%) | Univariate | |  | Multivariate |  |
| --- | --- | --- | --- | --- | --- | --- |
|  | |  | P ^a^ | HR (95 % CI) ^b^ | | *P* ^b^ |
| All patients | | 375 (100) |  |  | |  |
| Age (years) | |  |  |  | |  |
| ≤ 56 (median) | | 207 (55.2) |  |  | |  |
| ＞56 (median) | | 168 (44.8) | 0.255 |  | |  |
| FIGO Stage | |  |  |  | |  |
| Early (I+II) | | 58 (15.5) |  | 1.000 | |  |
| Late (III+IV) | | 317 (84.5) | **0.021** | 1.655 (1.061-2.581) | | **0.026** |
| Ascites | |  |  |  | |  |
| Absence | | 51 (13.6) |  |  | |  |
| Present | | 324 (76.4) | 0.165 |  | |  |
| Residual tumor (cm) | |  |  |  | |  |
| ≤1 | | 326 (86.9) |  |  | |  |
| ＞1 | | 49 (13.1) | 0.050 |  | |  |
| FBP1 expression status | |  |  |  | |  |
| Positive | | 177 (47.2) |  | 1.000 | |  |
| Negative | | 198 (52.8) | **0.000** | 0.582 (0.436-0.777) | | **0.000** |
| C-MYC expression status | |  |  |  | |  |
| Negative | | 206 (54.9) |  | 1.000 | |  |
| Positive | | 169 (45.1) | **0.005** | 1.402 (1.056-1.862) | | **0.028** |
| STAT3 expression status | |  |  |  | |  |
| Negative | | 309 (82.4) |  |  | |  |
| Positive | | 66 (17.6) | **0.009** |  | |  |
| p-STAT3 expression status | |  |  |  | |  |
| Negative | | 308 (82.4) |  |  | |  |
| Positive | | 67 (17.6) | **0.024** |  | |  |
| Chemotherapeutic response | |  |  |  | |  |
| Platinum sensitive | | 162 (43.2) |  | 1.000 | |  |
| Platinum resistant | | 213 (56.8) | **0.000** | 3.840 (2.787-5.289) | | **0.000** |

Kanplan-Meier survival analysis and Cox proportional hazards regression analysis

^a^without adjustment

^b^with adjustment for age, FIGO stage, residual tumor, FBP1 expressioin status and chemotherapeutic response
