# Supplementary material for: FBP1 regulates proliferation, metastasis, and chemoresistance by participating in C-MYC/STAT3 signaling axis in ovarian cancer
Source: Oncogene. 2021 Aug 6;40(40):5938–49. doi: 10.1038/s41388-021-01957-5 (PMC8497274; doi:10.1038/s41388-021-01957-5)
Supplement: Supplementary file 15 — Table S2 [file 41388_2021_1957_MOESM15_ESM.docx]

**Supplementary Table 2. The relationship between clinicopathological characteristics and FBP1, C-MYC, STAT3 and p-STAT3 expression in ovarian cancer patients**

| Prognostic factors | FBP1-low（%） | FBP1-high（%） | *P* ^a^ | C-MYC- low（%） | C-MYC- high（%） | *P* ^a^ | STAT3- low （%） | STAT3- high （%） | *P* ^a^ | p-STAT3 -low （%） | p-STAT3-high（%） | *P* ^a^ |
| --- | --- | --- | --- | --- | --- | --- | --- | --- | --- | --- | --- | --- |
| Age |  |  | 0.756 |  |  | 0.252 |  |  | 0.056 |  |  | 0.788 |
| ≤56 (median) | 96 (54.2) | 111 (56.1) |  | 108 (52.4) | 99 (58.6) |  | 178 (57.6) | 29 (43.9) |  | 171 (55.5) | 36 (53.7) |  |
| ＞56 (median) | 81 (45.8) | 87 (43.9) |  | 98 (47.6) | 70 (41.4) |  | 131 (42.4) | 37 (56.1) |  | 137 (44.5) | 31 (46.3) |  |
| FIGO Stage |  |  | 0.568 |  |  | 0.776 |  |  | 0.573 |  |  | 0.094 |
| Early (I+II) | 25 (14.1) | 33 (16.7) |  | 33 (16.0) | 25 (14.8) |  | 46 (14.9) | 12 (18.2) |  | 43 (14.0) | 15 (22.4) |  |
| Late (III+IV) | 152 (85.9) | 165 (83.3) |  | 173 (84.0) | 144 (85.2) |  | 263 (85.1) | 54 (81.8) |  | 265 (86.0) | 52 (77.6) |  |
| Ascites |  |  | **0.016** |  |  | **0.016** |  |  | 0.992 |  |  | 0.437 |
| Absence | 16 (9.0) | 35 (17.7) |  | 36 (17.5) | 15 (8.9) |  | 42 (13.6) | 9 (13.6) |  | 40 (13.0) | 11 (16.4) |  |
| Present | 161 (91.0) | 163 (82.3) |  | 170 (82.5) | 154 (91.1) |  | 267 (86.4) | 57 (86.4) |  | 268 (87.0) | 56 (83.6) |  |
| Residual tumor(cm) |  |  | **0.000** |  |  | 0.281 |  |  | 0.687 |  |  | 0.163 |
| ≤1 | 141 (79.7) | 185 (93.4) |  | 183 (88.8) | 143 (84.6) |  | 267 (86.4) | 59 (89.4) |  | 264 (85.7) | 62 (92.5) |  |
| ＞1 | 36 (20.3) | 13 (6.6) |  | 23 (11.2) | 26 (15.4) |  | 42 (13.6) | 7 (10.6) |  | 44 (14.3) | 5 (7.5) |  |
| Chemotherapeutic response |  |  | **0.037** |  |  | 0.061 |  |  | 0.273 |  |  | 0.341 |
| Platinum sensitive | 66 (37.3) | 96 (48.5) |  | 98 (47.6) | 64 (37.9) |  | 138 (44.7) | 24 (36.4) |  | 137 (44.5) | 25 (37.3) |  |
| Platinum resistant | 111 (62.7) | 102 (51.5) |  | 108 (52.4) | 105 (62.1) |  | 171 (55.3) | 42 (63.6) |  | 171 (55.5) | 42 (62.7) |  |
| Recurrence |  |  | **0.001** |  |  | 0.051 |  |  | 0.206 |  |  | 0.205 |
| Absence | 48 (27.1) | 86 (43.4) |  | 83 (40.3) | 51 (30.2) |  | 115 (37.2) | 19 (28.8) |  | 115 (37.3) | 19 (28.4) |  |
| Present | 129 (72.9) | 112 (56.6) |  | 123 (59.7) | 118 (69.8) |  | 194 (62.8) | 47 (71.2) |  | 193 (62.7) | 48 (71.6) |  |

Two-sided χ2 test or Fisher’s exact test for distributions between negative and positive expression of FBP1, C-MYC, STAT3, p-STAT3.
