# Supplementary material for: FBP1 regulates proliferation, metastasis, and chemoresistance by participating in C-MYC/STAT3 signaling axis in ovarian cancer
Source: Oncogene. 2021 Aug 6;40(40):5938–49. doi: 10.1038/s41388-021-01957-5 (PMC8497274; doi:10.1038/s41388-021-01957-5)
Supplement: Supplementary file 16 — Table S3 [file 41388_2021_1957_MOESM16_ESM.docx]

**Supplementary Table 3. The primer sequences of ChIP assay**

| Distance from FBP1(bp) | Number | Primer |
| --- | --- | --- |
| 3000-2700 | 1-F | CAAGTAAAGGGGCACACTC |
|  | 1-R | GCCTGGCCAACATGGTGAAA |
| 2700-2400 | 2-F | TGGTCTTGAACTCCTGACCT |
|  | 2-R | CACCTGAGGTTGGGAGCTCG |
| 2400-2100 | 3-F | ATCTGCCCGCCTCGGCCTC |
|  | 3-R | TGTGCCCAGGGTTGTCAGAT |
| 2100-1800 | 4-F | GGAATCTGCACCCCAGGT |
|  | 4-R | TTAGGAGCTAAGTGTGGG |
| 1800-1500 | 5-F | TTGTCAGTCCTGCCTCCAGC |
|  | 5-R | GAAGAGAGGCATGGTCTACCCT |
| 1500-1200 | 6-F | TTCTCCAAACTCTCCAGT |
|  | 6-R | GCCTCAGTAAAAGCTTCT |
| 1200-900 | 7-F | CTCTGCTCACCCTCTTCT |
|  | 7-R | GCTCCACCGCCCGCATCC |
| 900-600 | 8-F | CCTCTGGCCTTTGTGTGG |
|  | 8-R | TGGCTTTAGCTCTGCAGC |
| 600-300 | 9-F | GGCCATGTGGACTGGCTGCGGGTTT |
|  | 9-R | CCAGACCGCGGCTCCGCC |
| 300-0 | 10-F | TCCGCGGGGTAGGCGGGGCG |
|  | 10-R | GCTTGAACCGGGTAGAGCGC |
