# Supplementary material for: FBP1 regulates proliferation, metastasis, and chemoresistance by participating in C-MYC/STAT3 signaling axis in ovarian cancer
Source: Oncogene. 2021 Aug 6;40(40):5938–49. doi: 10.1038/s41388-021-01957-5 (PMC8497274; doi:10.1038/s41388-021-01957-5)
Supplement: Supplementary file 17 — Supplementary Figure Legends [file 41388_2021_1957_MOESM17_ESM.docx]

**Supplementary Figure Legends**

**Supplementary Figure 1. The association between FBP1 expression and survival of ovarian cancer patients.**

1. Kaplan-Meier overall survival and progression-free survival curve (log-rank tests) in the patients with high and low expression of FBP1.
2. Kaplan-Meier overall survival and progression-free survival curve of in stage III-IV patients with high and low expression of FBP1.

**Supplementary Figure 2. RNA sequencing after upregulation of FBP1**

1. Immunoblotting analysis of background expression levels of FBP1 in 12 ovarian cancer cell lines.
2. Representative image of established stable cell lines showed that FBP1-overexpressing vector were constructed and stably expressed in A2780/SKOV3 cells.
3. GSEA in FBP1-overexpressing and control A2780 cells. The signature was defined by genes with significant expression changes.
4. A heat-map display shows the FBP1-altered genes involved in metastasis, oxidative phosphorylation, cell apoptosis and response to cisplatin. Gene expression profiling was performed by RNA sequencing.

**Supplementary Figure 3. Influence of upregulation of FBP1 on cell proliferation, colony formation, and metastasis ability in *vitro*.**

1. Enhancement of FBP1 expression suppressed the proliferation of ovarian cancer A2780/SKOV3 cell lines. Cell viability determined by CCK8 assay.

**(B-C)** The colony-forming assays showed that overexpression of FBP1 suppressed cell proliferation in A2780/SKOV3 cells.

**(D-E)** Representative image (left panel) and quantitative analysis (right panel) of enhancement of FBP1 expression suppressed the invasiveness of ovarian cancer A2780/SKOV3 cell lines as detected by Transwell assay.

**(F-G)** Representative image (left panel) and quantitative analysis (right panel) of enhancement of FBP1 expression suppressed the migration of ovarian cancer A2780/SKOV3 cell lines as detected by scratching assay.

**(H)** Western blotting analysis of indicated proteins in FBP1-overexpressing and control A2780/SKOV3 cells. * *P* < 0.05, ** *P* < 0.01.

**Supplementary Figure 4. FBP1 regulates ovarian cancer cell colony formation, metastasis ability and chemosensitivity in *vitro*.**

1. Representative image of established stable cell lines showed that FBP1-silencing vector were constructed and down-expressed in OVCA433/ OVCA420 cells.

**(B-C)** The colony-forming assays showed that silencing of FBP1 promoted cell proliferation in OVCA433/ OVCA420 cells.

**(D-E)** Representative image and quantitative analysis of enhancement of FBP1 expression promoted the metastasis of ovarian cancer OVCA433/ OVCA420 cell lines as detected by Transwell assay.

**(F-G)** CCK-8 assays showed the effect of empty vector and FBP1 silencing on the chemosensitivity of ovarian cancer cells to the cytotoxic effect of cisplatin. * *P* < 0.05, ** *P* < 0.01.

**Supplementary Figure 5. Influence of upregulation of FBP1 on cell gluconeogenesis in *vitro*.**

1. Enhancement of FBP1 expression inhibited the glucose uptake dramatically in A2780 and SKOV3 cells when compared with controls.
2. Enhancement of FBP1 expression decreased the lactate production dramatically in A2780 and SKOV3 cells when compared with controls.
3. Enhancement of FBP1 expression decreased the ATP production dramatically in A2780 and SKOV3 cells when compared with controls.
4. Enhancement of FBP1 expression decreased the extracellular acidification rate (ECAR) dramatically in A2780 and SKOV3 cells when compared with controls. Error bars = 95% CIs.
5. Enhancement of FBP1 expression decreased the e oxygen consumption rate (OCR) dramatically in A2780 and SKOV3 cells when compared with controls. Error bars = 95% CIs.
6. Western blotting analysis of indicated proteins in FBP1-overexpressing and control A2780/SKOV3 cells. The experiments were repeated three times, and a representative experiment is shown.
7. Representative image of PET-CT scan of FBP1-overexpressing and controlled A2780/SKOV3 cells injected mice before the administration of cisplatin (left panel) and average SUV of nude mice bearing tumors before the administration of cisplatin (right panel). ** *P* < 0.01.

**Supplementary Figure 6.** Immunohistochemical staining of related molecules in the FBP1-overexpressing and control tissues.

Immunohistochemical staining of Ki67, E-cadherin, Caspase3, SOX2 and NANOG in the FBP1-overexpressing and control tissues.

**Supplementary Figure 7. SDS-PAGE result of mass spectrum analysis in A2780 cells with high expression of FBP1.**

1. SDS-PAGE analysis with Coomassie brilliant blue staining showed the proteins pulled down by FBP1 from A2780 cells. The arrow indicates the possible STAT3 in the FBP1-bound complex, the lane with the FBP1-bound complex was excised and subjected to mass spectrometry.
2. Western blotting analysis of STAT3 and phosphorylated STAT3 (p-STAT3) in FBP1-overexpressing and control A2780/SKOV3 cells. Representative data from three repeated experiments is shown.
3. Immunohistochemical staining of FBP1, STAT3 and p-STAT3 in the FBP1-overexpressing and control tissues.

**Supplementary Figure 8. FBP1 G260R mutant directly interacts with STATS and inhibits STAT3 expression and phosphorylation in ovarian cancer cells**

**(A)** Interaction between FBP1 G260R and STAT3 detected by Co-immunoprecipitation assay.

**(B)** Induction of FBP1 or FBP1 G260R significantly changed the distribution and expression of STAT3 and p-STAT3 (Tyr705) protein in the nucleus and cytoplasm.

**(C)** Interaction between FBP1 G260R and STAT3 in the nucleus and cytoplasm detected by Co-immunoprecipitation assay.

**(D)** Interaction between FBP1 G260R and STAT3 by FRET-FLIM upon transient coexpression in A2780 and SKOV3 cells. FE, FRET efficiency. Asterisks indicate a statistically significant difference (**, *P* value < 0.01), according to a Student’s t test.

**(E)** Representative immunofluorescence staining (×1000) images showing that FBP1 G260R inhibited the expression of STAT3 in the cell nucleus (red). Blue dye (DAPI) indicates the nucleus.

**Supplementary Figure 9. FBP1 G260R mutant inhibits cell proliferation, glycolysis, metastasis and chemosensitivity in ovarian cancer cells.**

1. Representative image of established stable cell lines showed that FBP1 G260R-overexpressing vector were constructed and stably expressed in A2780/SKOV3 cells.
2. FBP1 enzymatic activity in the A2780 and SKOV3 cell lysates.
3. Representative images and quantitative analysis of CCK-8 Kit assay rate showed the changes of cell proliferation rate after upregulated FBP1 G260R as well as STAT3 at low glucose level.
4. The glucose uptake was tested after upregulated FBP1 G260R as well as STAT3.
5. The lactate production was tested after upregulated FBP1 G260R as well as STAT3.
6. Quantitative analysis of colony formation rate showed the changes of cell colonies after upregulated FBP1 G260R as well as STAT3.
7. Quantitative analysis of invaded cells showed the changes of cell invasion by using Transwell assay after upregulated FBP1 G260R as well as STAT3.
8. Representative images of the relative protein expression detected by Western blotting in rescue experiment.
9. Quantitative analysis of apoptotic cells showed the changes of the cisplatin-induced apoptosis detected by flow cytometry after upregulated FBP1 G260R as well as STAT3.
10. Representative images of the relative protein expression detected by Western blotting in rescue experiment with cisplatin treatment. ** *P* < 0.01.

**Supplementary Figure 10. The anti-cancer effect of FBP1 may be achieved by inhibiting the expression of STAT3.**

**(A-B)** Representative images and quantitative analysis of colony formation rate showed the changes of cell colonies after upregulated FBP1 as well as STAT3.

**(C-D)** Representative images and quantitative analysis of invaded cells showed the changes of cell invasion by using Transwell assay after upregulated FBP1 as well as STAT3.

**(E-F)** The glucose uptake, lactate production was tested after upregulated FBP1 as well as STAT3.

**(G)** Representative images of the relative protein expression detected by Western blotting in rescue experiment.

**(H)** Quantitative analysis of apoptotic cells showed the changes of the cisplatin-induced apoptosis detected by flow cytometry after upregulated FBP1 as well as STAT3.

**(I)** Representative images of the relative protein expression detected by Western blotting in rescue experiment with cisplatin treatment. * *P* < 0.05, ** *P* < 0.01.

**Supplementary Figure 11. The anti-cancer effect of FBP1 may be achieved by inhibiting the expression of STAT3 *in vivo.***

1. Representative image of nude mice bearing tumors formed by SKOV3 cells.
2. The average tumor volume with or without cisplatin treatment formed by SKOV3 cells.
3. Representative image of nude mice bearing tumors formed by A2780 cells.
4. The average tumor volume with or without cisplatin treatment formed by A2780 cells.

**Supplementary Figure 12. Tumor-suppressive properties mediated by C-MYC silencing are attenuated when FBP1 expression is abrogated.**

**(A)** Immunoblotting analysis of background expression level of FBP1 and C-MYC in four ovarian cancer cell lines.

**(B)** Immunoblotting analysis of knockdown of c-myc in A2780/SKOV3 cells.

**(C)** Representative images and quantitative analysis of colony formation rate showed the changes of cell colonies after knockdown of c-myc as well as FBP1.

**(D)** Representative images and quantitative analysis of invaded cells showed the changes of cell invasion by using Transwell assay after knockdown of c-myc as well as FBP1.

**(E-F)** The glucose uptake and lactate production was tested after silencing c-myc as well as FBP1.

**(G)** Quantitative analysis of apoptotic cells showed the changes of the cisplatin-incuced apoptosis detected by flow cytometry after knockdown of C-MYC as well as FBP1.

**(H-I)** Representative images of the relative protein expression detected by Western blotting in rescue experiment with or without cisplatin treatment. ** *P* < 0.01.
