# Supplementary material for: FBP1 regulates proliferation, metastasis, and chemoresistance by participating in C-MYC/STAT3 signaling axis in ovarian cancer
Source: Oncogene. 2021 Aug 6;40(40):5938–49. doi: 10.1038/s41388-021-01957-5 (PMC8497274; doi:10.1038/s41388-021-01957-5)
Supplement: Supplementary file 18 — Supplemental Material and Methods [file 41388_2021_1957_MOESM18_ESM.docx]

**Supplemental Materials**

**Material and Methods**

**Cell lines and culture**

The established human ovarian cancer cell lines were obtained from the Cell Bank of the Chinese Academy of Science. All cells were maintained in Dulbecco’s modified Eagle’s medium (DMEM, HyClone, Thermo Scientific, USA) supplemented with 10% fetal bovine serum (Gibco, Life technologies, USA), 100 U/ml penicillin (Biowest, Nuaillé, France), and 100 U/ml streptoc-mycin (Biowest, Nuaillé, France) and incubated at 37°C in a humidified atmosphere with 7% CO_2_.

**Immunohistochemistry assay**

The 10×12 tissue microarray (TMA) was made by FUSCC Tissue Bank. IHC was performed on 7-μm-thick TMA sections using the antibody against FBP1 (ab196556, rabbit polyclonal antibody, Abcam, 1:200 dilution)，C-MYC (ab32, mouse monoclonal antibody, Abcam, 1:300 dilution), STAT3 (#9139, mouse monoclonal antibody, Cell Signaling Technology, 1:500 dilution), phospho-STAT3 (#9145, rabbit monoclonal antibody, Cell Signaling Technology, 1:500 dilution). Each case has two cores made from separate sources to preclude the heterogeneity of tumors. A known positive case sample was included as a positive control, and the primary antibody was replaced with non-immune mouse/rabbit serum for negative control. The immunoreactive Score (IRS) was multiplicity of the staining intensity and positive cancer percentage. Finally, the assessment of the protein expression was defined as negative (≤1+) and positive (>2+to ≤3+).

**Whole-body ^18^F-FDG positron emission tomography/computed tomography (PET/CT)**

Whole-body FDG PET/CT was performed as previously described (24). Briefly, 18F-FDG was automatically made by a cyclotron (Siemens CTI RDS Eclipse ST) using an Explora FDG4 module. Patients had been fasting for more than 6 hours. Scanning started 1 hour after intravenous injection of the tracer (7.4 MBq/kg). The images were acquired on a Siemens biograph 16HR PET/CT scanner with a transaxial intrinsic spatial resolution of 4.1 mm. CT scanning was first initiated from the proximal thighs to the head, with 120 kV, 80–250 mA, pitch 3.6, and rotation time of 0.5 seconds. Image interpretation was carried out on a multimodality computer platform (Syngo; Siemens). Quantification of metabolic activity was acquired using the SUV normalized to body weight, and the SUVmax for each lesion was calculated.

**Plasmids construction and viral infection**

The recombinant plasmid pENTER-FBP1 and pENTER-STAT3 containing human full cDNA sequence of FBP1 and STAT3 was purchased from Vigene Biosciences (Jinan, China). Then the cDNA sequence of FBP1 and STAT3 was subcloned into lentivirus vector. The recombinant plasmid pCDH-FBP1 G260R was purchased from Hanyin Biotechnology Limited Company (Shanghai, China). Lentivirus was produced by co-transfecting 263T cells with pRSV-Rev, pMD2.G, pMDLg/pRRE and pCDH-puro expression vectors. Virus was harvested after 48 hours by filtering the virus-containing medium through 0.45 μM Steriflip filter (Millipore). A2780, SKOV3 and A2780 cisplatin-resistance cells (A2780/DDP or A2780·cis) were infected by incubating cells with medium containing indicated virus and 8 μg/mL polybrene (Sigma) for 24 hours. Established stable cell lines expressing FBP1 were constructed as above. Control cell lines were generated by infection with viruses containing the empty vector by following the same protocol. In addition, the human short hairpin RNA (shRNA) of FBP1 was purchased from Hanyin Biotechnology Limited Company (Shanghai, China) and interfering RNA (siRNA) targeting C-MYC were purchased from Guangzhou Ribobio. The shRNA sequence of FBP1 is as followed FBP1-sh1：5-ggacaaggatgtgaagata-3，FBP1-sh2：5-ctgagtacatccagaggaa-3， FBP1-sh3：5-ggaaggaggccgtgttaga-3.

The promoter sequences of FBP1 (-3000~+84 bp) were cloned from genomic DNA prepared from A2780 cells using Genomic DNA Extraction Kit (TIANGEN BIOTECH CO.LID, Beijing). Then, the promoter sequences of FBP1 were subcloned into pGL3-basic vector and established the recombinant plasmid, pGL-FBP1-promoter. The pGL-FBP1-promoter recombinant plasmid was co-transfected with phRl-TK vector into A2780 and SKOV3 cells using Lipofectamine 2000 according to the manufacturer's instructions. The potential transcript factor binding sites in the promoter region of FBP1 was predicted using GeneCards software (www.genecards.org) and UCSC Genome Bioinformatics Site (http://genome.ucsc.edu/).

**TCGA RNA-seq data analysis**

Raw RNAseq data for 564 ovarian serous adenocarcinoma were downloaded from cBioPortal (http://www.cbioportal.org/index.do) originated from the TCGA project. Data was analyzed for differential gene expression. Fold expression change for FBP1 were exported.

**RNA-seq data analysis**

Total RNA (1μg) was isolated from A2780 cells and treated with VAHTS mRNA Capture Beads (Vazyme, Nanjing, China) to enrich polyA+ RNA before constructing the RNA libraries. RNA library preparation was performed by using VAHTS mRNA-seq v2 Library Prep Kit for Illumina (Vazyme, Nanjing, China). Paired-end sequencing was performed with Illumina HiSeq 3000 at RiboBio Co., Ltd (Guangzhou, China). For computational analysis of RNA-seq data, sequencing reads were aligned using the spliced read aligner HISAT2, which was supplied with the Ensembl human genome assembly (Genome Reference Consortium GRCh38) as the reference genome. Gene expression levels were calculated by the FPKM (fragments per kilobase of transcript per million mapped reads). Gene Set Enrichment Analysis (GSEA) was used for gene functional annotation.

**Human Ovarian Organoids**

Human Ovarian Organoids Immunofluorescence assay was performed as previously described. Cisplatin-sensitive organoids in our study were generated from cisplatin-sensitive ovarian cancer patients’ tissues in the first surgery. The cisplatinresistant PDOs were gained from ovarian cancer tissues of the cisplatin-resistant patients, who underwent reoperation after failure of cisplatin-based chemotherapy. Then the PDOs were cultured as described below and the drug resistance tests were performed with the treatment of cisplatin for 21 days. For PDOs generation, fresh tumor tissues were immediately transported to laboratory in Advanced DMEM/F12 supplement with 1 % penicillin streptomycin after obtained in the surgery. Tissues were diced into approximately 2-3 mm sections and then digested in 37°C for 1 hrs. Digestion solution was made of Advanced DMEM/F12 containing type IV Collagenase (Sigma-Aldrich; catalog number C9407). The digested sample was filtered through a 70-μm filter (Falcon; catalog number 352350). The cell suspension was then spun at 1000 RPM for 5 min to create a cell pellet. The pellet was washed with red blood for 2-3 times. Cells of solid tumor were then mixed with growth factor reduced Matrigel (Corning; catalog number CB-40230C), and cells concentration was 10,000/50 μl~20,000 cells/50 μl. Once the Matrigel was solidified, 500 μL of general culture medium was added. Cultures were overlaid with medium containing specific growth factors as the method of Willert [44] with a few modifications. PDOs were kept in a humidified atmosphere of 5% CO2 and 95% air at 37 °C, and medium was changed every 2~3 days

**Western blotting assay**

Antibodies against Bcl-2, BAX, GLUT4, HK2, MMP3, E-cadherin were from proteintech. Antibodies against C-MYC, FBP1 were from abcam. The antibody to β-Actin, STAT3 and phosphor-STAT3 (Ty705) were purchased from Cell Signaling Technology. All the primary antibodies were used at 1:1000 dilutions and secondary antibodies at 1:5000 dilutions. Western blot analysis was performed to determine the expression levels of various proteins in cells. Cells were rinsed in ice-cold 1×PBS twice, and lysed with RIPA lysis buffer containing protease inhibitors (150 mM NaCl, 50 mM Tris–HCl, pH 8.0, 0.05 MEDTA, 1% Triton X-100, 0.1%SDS and 0.005× protease inhibitor cocktail) for 30 min on ice, then centrifuged at 12,000g for 15 min at 4℃. The total protein concentration was determined by BCA protein assay kit (Beyotime). Equal amounts (30 mg per load) of protein samples were resolved on 10% SDS-PAGE electrophoresis and transferred on to polyvinylidene fluoride (PVDF) membranes (Merck Millipore, Darmstadt, Germany). The blots were blocked in 8% non-fat milk and incubated with primary antibodies with b-actin used as an internal control, followed by incubation with secondary antibodies conjugated with horseradish peroxidase (HRP). Immunoreactivity was detected with the chemiluminescent reagents (Millipore). Three independent experiments were done for final analyses.

**Reverse transcription quantitative real-time polymerase chain reaction (RT-qPCR)**

For the tissue samples, total RNA was extracted with TRIzol (Invitrogen) according to the manufacturer’s instructions. For the blood samples, 0.5 ml serum was prepared by centrifuging at 4,000 rpm at 4℃ and then mixed with 1 ml TRIzol. All RNAs were then reverse transcribed into cDNAs using the PrimeScript RT-PCR kit (TaKaRa, Japan) following the manufacturer’s instructions. Oligonucleotide primer pairs for FBP1 and GAPDH are described as followed: FBP1, F: 5’-CTACGCCAGGGACTTTGACC-3’, R: 5’-GGCCCCATAAGGAGCTGAAT-3’; GAPDH, F: 5’-GGCCTCCAAGGAGTAAGACC-3’, 5’-R: CAAGGGGTCTACATGGCAAC-3’. All amplifications and detections were carried out in the Applied Biosystems Prism 7900 system (Applied Biosystems, Foster City, CA) using the SYBR green QPCR kit (TaKaRa) and the following program: 1 cycle of 30 second at 95℃ followed by 40 cycles of (5 sec at 95℃ 20 second at 60℃), followed by a 30-min melting curve collection, which was used to verify the primer dimers. Statistical analyses were performed using the 2^-△△CT^ relative quantification method. The assays were repeated three times in triplicate.

**Immunofluorescence assay**

Immunofluorescence staining was done according to the published protocol. Primary antibodies against FBP1 (ab196556, rabbit polyclonal antibody, Abcam), STAT3 (#9139, mouse monoclonal antibody, Cell Signaling Technology), and p-STAT3 (#9145, rabbit monoclonal antibody, Cell Signaling Technology) were obtained respectively. DNA dye 4′,6-diamidino-2-phenylindole (DAPI) was obtained from Molecular Probes. The secondary antibodies used were either FITC-conjugated against rabbit IgG or Texas red–conjugated against mouse IgG (ShareBio Technology). All stained cells were examined and photographed with an Olympus FV500 confocal fluorescence microscope.

**Luciferase reporter assay**

Luciferase reporter assay was performed as previously described. Human FBP1 gene promoter region was inserted into a pGL3 basic vector as pGL3-FBP1-Promoter. One hundred nanogram (ng) of constructed plasmid and 7 ng renilla luciferase control plasmid were transfected into cells silencd by shc-myc in six-well plates. Forty-eight hours later, luciferase activities were measured using the Dual Luciferase Assay Kit (Promega, Madison, WI, USA). Renilla luciferase were used to normalize reporter luciferase activities,

**Chromatin Immunoprecipitation (ChIP) assay**

ChIP Assays were performed using Pierce Agarose ChIP Kit (Thermo, #27177). Briefly, A2780 were cross-linked by 1% formaldehyde for 10 min at 37 °C. The cross-linking reaction was quenched by glycine and cells were lysed in SDS buffer containing protease inhibitor cocktail. Cell lysates were sonicated to shear chromatin DNA into fragments with 200–1,000 base pairs in size and then subjected to immunoprecipitation with 4μl IgG (Cell Signaling Technology), 7μl C-MYC (ab32, mouse monoclonal antibody, Abcam) or STAT3 (#9139, mouse monoclonal antibody, Cell Signaling Technology) or 2μl Polymerase II (Imgenex) antibodies. After washing with a series of low and high salt concentration washing buffers, immunoprecipitated DNA fragments were de-crosslinked at 77 °C in high salt condition, purified using QIAquick PCR purification kit (Qiagen), and then analysed by qRT-PCR.

Using the GAPDH promoter primers (Kit) confirmed the effectiveness of conventional PCR chip results. The correct chip results should be that only the input and RNApolII samples will have positive results, which could be shown as a 300bp band PCR, and the other three groups (IgG, C-MYC and STAT group 3) appeared no band. In the ORF region of human FBP1 gene, which located within upstream 3000bp long of the target gene, a pair of primers was designed by using of Primer7.0 every 300bp or so.

**Subcellular fractionation**

By following the manufacturer’s protocol, cytosolic and nuclear fractionation of indicated cells were performed using nuclear and cytoplasmic extraction Kit (Tiangen Biotech, China).

**Immunoprecipitation**

Cells were collected and lysed in RIPA lysis buffer (Beyotime) and protease inhibitor cocktail (Roche Diagnostics). 2 mg whole cell lysates were pre-cleared with 30 μL protein G beads (Life Technologies), and then add 2 μg isotype-matched IgG control or indicated antibodies incubating for 2 hours on a rocking platform. The immunoprecipitates were collected by centrifugation and then resolved by SDS-PAGE.

**GST pull down assay**

Recombinant plasmids pGEX-4T3, which encode indicated GST-tagged (stat3 functional regions 1-6) proteins relatively, were separately transformed into BL21-DE3 chemically competent E. coli (TIANGEN Biotech, China). Protein expression was induced by adding IPTG (Takara Biotechnology, Shiga, Japan) (1 mM) into LB media, then rocked at 16 °C for 20 hours. Bacterial pellets were collected and re-suspended with PBS buffer supplemented with 100 μg/mL protease inhibitor cocktail (Roche Diagnostics) and were sonicated on ice with 15-second pulses at high intensity（35Hz）(BioTeK). Bacteria were broken down in the ultrasonic environment. The specific time depends on the amount, weight and suspension of the bacteria to be clarified. Then centrifuge and collect the supernatants mixture of protein. Add 20 μL Glutathione Sepharose 4B to the EP tube, and rinsed with the pre-cooled PBS for 3 times and then centrifuged 12000 g for, 20 s and discard supernatants. Cells were collected and lysed in RIPA lysis buffer (Beyotime) and protease inhibitor cocktail (Roche Diagnostics). One mL Mixed dialysis products and cell lysates 1ml were added to the EP tube containing 20 L Glutathione Sepharose 4B and incubated at 4℃ for 4 h. Then products were resolved by SDS-PAGE.

**Protein purification**

Recombinant plasmids pGEX-4T3 and pET-28a, which encode indicated GST-tagged (stat3 functional regions 1-6) and His-tagged (fbp1 exon 1-7) proteins relatively, were separately transformed into BL21-DE3 chemically competent E. coli (TIANGEN Biotech, China). Protein expression was induced by adding IPTG (Takara Biotechnology, Shiga, Japan) (1 mM) into LB media, then rocked at 16 °C for 20 hours. Bacterial pellets were collected and re-suspended with PBS buffer supplemented with 100 μg/mL protease inhibitor (Roche Diagnostics) and were sonicated on ice with 15-second pulses at high intensity（35Hz）(BioTeK). Then centrifuge and collect the supernatants mixture of protein for further purification.

GST-tagged and His-tagged proteins were further purified using GST or Ni-NTA Sefinose Resin kit (Sangon Biotech, China) by following the manufacturer’s protocol. Briefly, add above mixture of protein extract with Binding Buffer to columns filled with resin, which have a high-affinity to GST-tagged or His-tagged recombinant proteins. Eliminate non-specific binding proteins by washing the resin until the absorbance of the flow-through fraction at 280 nm approaches baseline. Then elute recombinant proteins from the resin with Elution Buffer. Monitor protein elution by measuring the absorbance of the fractions at 280 nm. Finally, the eluted proteins were analyzed by SDS-PAGE.

**Protein mass spectrometry assay**

When the gel containing target fragment was cut, added 100 mM ammonium bicarbonate/acetonitrile (1:1, V/V) and incubate with vortexing to destain the gel pieces (may repeat several times according to the staining intensity). Added 500 µL ACN into the tube and incubate until the gel pieces shrink and then discard all the liquid. Added the trypsin buffer (13 ng/µL, in 10 mM ammonium bicarbonate containing 10% (V/V) acetonitrile) to cover the dry gel pieces and keep it on ice for about 30 min. Added more trypsin buffer to make sure the gel pieces being completely covered with trypsin buffer. The samples were incubated at 37 ºC overnight for tryptic digestion.

Each sample was resuspended with 30μl solvent A, respectively (A: water with 0.1% formic acid; B: ACN with 0.1% formic acid), separated by nanoLC and analyzed by on-line electrospray tandem mass spectrometry. The experiments were performed on an Easy-nLC 1000 system (Thermo Fisher Scientific, MA, USA) connected to a Q-Exactive mass spectrometer (Thermo Fisher Scientific, MA, USA) equipped with an online nano-electrospray ion source. A total of 10μl peptide sample was loaded onto the trap column (Thermo Scientific Acclaim PepMap C18, 100μm x 2cm), with a flow of 10μl/min for 3 min and subsequently separated on the analytical column (Acclaim PepMap C18, 75μm x 15cm) with a linear gradient, from 3% B to 32% B in 120min. The column was re-equilibrated at initial conditions for 10 min. The column flow rate was maintained at 300nL/min. The electrospray voltage of 2kV versus the inlet of the mass spectrometer was used. The mass spectrometer was run under data dependent acquisition mode, and automatically switched under MS and MS/MS mode. MS1 mass resolution was set as 35K with m/z 350-1550 and MS/MS resolution was set as 17.5K under HCD mode. The dynamic exclusion time was set as 20 seconds. Tandem mass spectra were processed by PEAKS Studio version 8.5 (Bioinfor Inc. CA). PEAKS DB was set up to search the UniProt human database (71544 entries) assuming the digestion enzyme Trypsin. PEAKS DB was searched with a fragment ion mass tolerance of 0.05 Da and a parent ion tolerance of 7.0 PPM. Oxidation (M), Deamidation (NQ), Acetylation (Protein N-term), were specified as variable modifications. Proteins were filter by -10log*P* peptide score above 20 and at least containing one unique peptide.

**Co-immunoprecipitation assay**

Purified FBP1 exon proteins 1 to 7 were incubated with Ni-NTA beads (BBI life science) on a rotator at 4°C for 1 hour (500 μg/ exon). Then purified proteins of functional regions1-6 of stat3 were equally (500 μg/ region) and separately added to each exon protein tube of fbp1 and mixed on a rotator at 4°C overnight. The immunoprecipitates were collected by centrifugation and then resolved by SDS-PAGE (15μL/load). Separated proteins were transferred on to polyvinylidene fluoride (PVDF) membranes (Millipore, Billerica, MA, USA) and incubated with GST-primary antibody (dilution 1:1000, proteintech), followed by incubation with secondary antibody conjugated with horseradish peroxidase (HRP). The protein bands were detected with the chemiluminescent reagents (Millipore).

**Glycolysis analysis**

Cells were plated in 6-well plates and cultured in DMEM medium without phenol red (Thermo Fisher Scientific). 24 hours after plasmid transfection or 48 hours after lentivirus infection, the spent medium was collected. Glucose concentration of the spent medium was measured using a Glucose (GO) Assay Kit according to the manufacturer’s instructions (Sigma-Aldrich). Glucose consumption was calculated by the difference of glucose concentration between the spent medium and unused medium.

**Oxygen consumption rate and extracellular acidification rate**

Cellular mitochondrial function was measured using the Seahorse XF Cell Mito stress test Kit and the Bioscience XF96 Extracellular Flux Analyzer, according to the manufacturer's instructions. The glycolytic capacity was determined using the Glycolysis Stress Test Kit as per the manufacturer's instructions. Briefly, 4 ×10^4^ cells were seeded onto 96-well plates and incubated overnight. After washing the cells with Seahorse buffer (DMEM with phenol red containing 25 mmol/L glucose, 2 mmol/L sodium pyruvate, and 2 mmol/L glutamine), 175 mL of Seahorse buffer plus 25 mL each of 1 mmol/L oligomycin, 1 mmol/L FCCP, and 1 mmol/L rotenone was automatically injected to measure the oxygen consumption rate (OCR). Then, 25 mL each of 10 mmol/L glucose, 1 mmol/L oligomycin, and 100 mmol/L 2-deoxy-glucose were added to measure the extracellular acidification rate (ECAR). The OCR and ECAR values were calculated after normalization to the cell number and are plotted as the mean ± SD.

**Cell proliferation assay and cell viability assay**

Multiple cultures of ovarian cancer cells were plated in 96-well plates at a density of 1×10^3^ cells/well supplemented with 100μl maintenance medium to evaluate cell proliferation rate. Each day one set of cultures was collected and counted. Cell Counting Kit-8 (CCK-8) (Dojindo Laboratories, Kumamoto, Japan) was used to measure cell growth at 0–8 day and the number of viable cells was determined by measurement of absorbance at 470 nm by a Microplate Reader (Synergy H4, Bio-Tek). The proliferation rate equaled the experimental OD value/ the control OD value. Cell viability was also assessed by CCK-8. We plated 8×10^3^ cancer cells per well in 96-well plates. The next day, the cells were treated with various concentrations of cisplatin. Cell viability was then measured based on the above protocols. All experiments were done in triplicate.

**Colony formation assay**

Cells were plated in a six-well plate in triplicate. The cells were filled with fresh medium and allowed to grow for 2 weeks before being fixed with ice-cold methanol and stained with Crystal violet. The experiments were done at least three times for final analyses.

**Cell apoptosis analysis**

To detect apoptosis, adherent cells were incubated with cisplatin at different concentrations. After 48h, cells were collected, washed twice with cold 1×PBS, and resuspended in 200 μL binding buffer at density of 1 × 10^7^cells / mL. Then we stained cells with 5 μL Annexin-V and PI (BD Biosciences) using an apoptosis detection kit (BD Biosciences, San Jose, CA, USA) and subjected to analysis by flow cytometry (Cytomics FC 500 MPL, Beckman Coulter). The early apoptosis was determined based on the percentage of cells with Annexin V+/PI-, while the late apoptosis was that of cells with Annexin V+/PI+. The experiments were done at least three times for final analyses.

**Cell invasion and migration assay**

To test cell invasion and migration, a 24-well plate with two-chamber plate (BD Biosciences, San Jose, CA) and an 8-μm (pore size) polycarbonate filter between chambers were obtained. We planted ovarian cancer cells in upper chamber supplemented with medium of no serum and allowed cells to invade or migrate for 24 hours at 37°C toward medium with serum. The cells were then fixed in 30% Paraformaldehyde for 30 minutes and stained with crystal violet for 10 minutes. All cells were counted at ×200 magnification under a microscope. We randomly selected three visual fields and the average invasive cells were counted as those passed through the membrane separating the chamber. Three independent experiments were done for final analyses.

**Sphere formation**

A2780 and SKOV3 cell lines were plated in 96-well ultra-low attachment plates (Corning, NY, USA) in serum-free Dulbecco’s modified Eagle medium/F12 medium supplemented with 20% knockout Serum Replacement (Life Technologies), 20 ng/mL epidermal growth factor, and 10 ng/mL basic fibroblast growth factor, at a density of 500 or 1000 viable cells/well. Spheres (>100 cells) were counted for 1-3weeks.

**FRET-FLIM Imaging**

For FRET-FLIM experiments, donor proteins (fused to GFP) were expressed from vectors pCMV3-C-GFPSpark, and acceptor proteins (fused to RFP) were expressed from vector CMV3-C-OFPSpark. FRET-FLIM experiments were performed on a Leica TCS SMD FLCS confocal microscope excitation with WLL (white light laser) and emission collected by a SMD SPAD (single photon-sensitive avalanche photodiodes) detector. A2780 or SKOV3 cells transiently coexpressing donor and acceptor, as indicated in the figures, were visualized 36h after agroinfiltration. Accumulation of the GFP- and RFP-tagged proteins was estimated before measuring lifetime. The tunable WLL set at 489 nm with a pulsed frequency of 40 MHz was used for excitation, and emission was detected using SMD GFP/RFP Filter Cube (with GFP: 500–550 nm). The fluorescence lifetime shown in the figures corresponding to the average fluorescence lifetime of the donor (τ) was collected and analyzed by PicoQuant SymphoTime software. Lifetime is normally amplitude-weighted mean value using the data from the single (GFP-fused donor protein only or GFP-fused donor protein with free RFP acceptor or with noninteracting RFP-fused acceptor protein) or biexponential fit (GFP-fused donor protein interacting with RFP-fused acceptor protein). Mean lifetimes are presented as means ± SD based on more than 10 cells from at least three independent experiments. FRET efficiency was calculated according to the formula E = 1 − τDA/τD, where τDA is the average lifetime of the donor in the presence of the acceptor and τD is the average lifetime of the donor in the absence of the acceptor.

**Flow cytometry analysis**

ALDH enzymatic activity was analyzed with ALDEFLUOR Kit by (MoFlo XDP) according to the manufacturer's instruction (StemCell Technologies, Vancouver, Canada). Ovarian cancer cells were suspended in ALDEFLUOR assay buffer including ALDH substrate (BAAA, 1 μmol/ 1×10^6^ cells) and then incubated at 37°C for 30 minutes. An aliquot of each sample was treated with 50mmol/L diethylaminobenzaldehyde (DEAB), a specific ALDH enzyme inhibitor, as negative control for background fluorescence. The ALDH^+^ population was defined by cells with increased FITC signal, with gates determined by DEAB-treated cells.

**Animal studies**

Animal experiments were approved by the Ethics Committee at FUSCC. Briefly, Female BALB/c nude (Shanghai Slac Laboratory Animal Co. Ltd, 4-6 weeks) were subcutaneously and intraperitoneally injected with FBP1-overexpressing A2780 cells (5×10^6^ suspended in 0.1 mL PBS for each mouse) and FBP1-overexpressing SKOV3 cells (5×10^6^ suspended in 0.1 mL PBS for each mouse). As for intraperitoneally *xenograft* model, the mice were weighed every 3 days and sacrificed for detecting of ascites and the size, number, weight of celiac metastasis of different cell lines. For subcutaneously *xenograft* model, measurement of tumor growth with a digital caliper was done every 3 days. Tumor volumes were calculated by the dimensional size of each tumor with the following formula: V (volume) = L (length) × W (width) ^2^ ×0.72. Once reaching an average tumor volume of 100 mm^3^，before treated with cisplatin, all the mice were subjected to perform PET/CT scan. The glucose uptake of tumor was evaluated by the standard uptake value (SUV). Then they were intraperitoneally treated with cisplatin (5mg/kg) thereafter. Administration of vehicle or agents and measurement of tumor volume were done every 3 days. Animals were also subjected to fluorescence imaging. Finally, the mice were weighed and sacrificed, and the tumors were weighed and dissected. RT-PCR and IHC of xenograft tumor were done according to the protocol above.

**Bisulfite Treatment of DNA and Methylation-Specific PCR**

Genomic DNA prepared from ovarian cancer patients and health women tissues was bisulfite-treated using Zymo DNA Modification Kit (Zymo Research, Orange, Calif., USA) according to the protocol provided. Methylation-specific PCR (MSP) was performed for 40 cycles with annealing temperature at 62°C. Methylation-specific primers were: FBP1-MF 5Research, Orange, Calif., US and FBP1-MR 5nd FBP1-MR range CAAATACGAA-3′, and unmethylation-specific primers were: FBP1-UF 5specific primer GGTGGAGTTGTG-3′ and FBP1-UR 5 and FBP1-UR primer GGTGGA GTTGTG-5’.

**Supplementary Figure Legends**

**Supplementary Figure 1. The association between FBP1 expression and survival of ovarian cancer patients.**

1. Kaplan-Meier overall survival and progression-free survival curve (log-rank tests) in the patients with high and low expression of FBP1.
2. Kaplan-Meier overall survival and progression-free survival curve of in stage III-IV patients with high and low expression of FBP1.

**Supplementary Figure 2. RNA sequencing after upregulation of FBP1**

1. Immunoblotting analysis of background expression levels of FBP1 in 12 ovarian cancer cell lines.
2. Representative image of established stable cell lines showed that FBP1-overexpressing vector were constructed and stably expressed in A2780/SKOV3 cells.
3. GSEA in FBP1-overexpressing and control A2780 cells. The signature was defined by genes with significant expression changes.
4. A heat-map display shows the FBP1-altered genes involved in metastasis, oxidative phosphorylation, cell apoptosis and response to cisplatin. Gene expression profiling was performed by RNA sequencing.

**Supplementary Figure 3. Influence of upregulation of FBP1 on cell proliferation, colony formation, and metastasis ability in *vitro*.**

1. Enhancement of FBP1 expression suppressed the proliferation of ovarian cancer A2780/SKOV3 cell lines. Cell viability determined by CCK8 assay.

**(B-C)** The colony-forming assays showed that overexpression of FBP1 suppressed cell proliferation in A2780/SKOV3 cells.

**(D-E)** Representative image (left panel) and quantitative analysis (right panel) of enhancement of FBP1 expression suppressed the invasiveness of ovarian cancer A2780/SKOV3 cell lines as detected by Transwell assay.

**(F-G)** Representative image (left panel) and quantitative analysis (right panel) of enhancement of FBP1 expression suppressed the migration of ovarian cancer A2780/SKOV3 cell lines as detected by scratching assay.

**(H)** Western blotting analysis of indicated proteins in FBP1-overexpressing and control A2780/SKOV3 cells. * *P* < 0.05, ** *P* < 0.01.

**Supplementary Figure 4. FBP1 regulates ovarian cancer cell colony formation, metastasis ability and chemosensitivity in *vitro*.**

1. Representative image of established stable cell lines showed that FBP1-silencing vector were constructed and down-expressed in OVCA433/ OVCA420 cells.

**(B-C)** The colony-forming assays showed that silencing of FBP1 promoted cell proliferation in OVCA433/ OVCA420 cells.

**(D-E)** Representative image and quantitative analysis of enhancement of FBP1 expression promoted the metastasis of ovarian cancer OVCA433/ OVCA420 cell lines as detected by Transwell assay.

**(F-G)** CCK-8 assays showed the effect of empty vector and FBP1 silencing on the chemosensitivity of ovarian cancer cells to the cytotoxic effect of cisplatin. * *P* < 0.05, ** *P* < 0.01.

**Supplementary Figure 5. Influence of upregulation of FBP1 on cell glycolysis in *vitro*.**

1. Enhancement of FBP1 expression inhibited the glucose uptake dramatically in A2780 and SKOV3 cells when compared with controls.
2. Enhancement of FBP1 expression decreased the lactate production dramatically in A2780 and SKOV3 cells when compared with controls.
3. Enhancement of FBP1 expression decreased the ATP production dramatically in A2780 and SKOV3 cells when compared with controls.
4. Enhancement of FBP1 expression decreased the extracellular acidification rate (ECAR) dramatically in A2780 and SKOV3 cells when compared with controls. Error bars = 95% CIs.
5. Enhancement of FBP1 expression decreased the e oxygen consumption rate (OCR) dramatically in A2780 and SKOV3 cells when compared with controls. Error bars = 95% CIs.
6. Western blotting analysis of indicated proteins in FBP1-overexpressing and control A2780/SKOV3 cells. The experiments were repeated three times, and a representative experiment is shown.
7. Representative image of PET-CT scan of FBP1-overexpressing and controlled A2780/SKOV3 cells injected mice before the administration of cisplatin (left panel) and average SUV of nude mice bearing tumors before the administration of cisplatin (right panel). ** *P* < 0.01.

**Supplementary Figure 6. FBP1 regulates ovarian cancer cell colony formation, metastasis ability and chemosensitivity in *vitro*.**

**(A-B)** CCK-8 assays showed the effect of empty vector and FBP1 silencing on the chemosensitivity of ovarian cancer cells to the cytotoxic effect of cisplatin. * *P* < 0.05, ** *P* < 0.01.

**Supplementary Figure 7.** Immunohistochemical staining of related molecules in the FBP1-overexpressing and control tissues.

Immunohistochemical staining of Ki67, E-cadherin, Caspase3, SOX2 and NANOG in the FBP1-overexpressing and control tissues.

**Supplementary Figure 8. SDS-PAGE result of mass spectrum analysis in A2780 cells with high expression of FBP1.**

1. SDS-PAGE analysis with Coomassie brilliant blue staining showed the proteins pulled down by FBP1 from A2780 cells. The arrow indicates the possible STAT3 in the FBP1-bound complex, the lane with the FBP1-bound complex was excised and subjected to mass spectrometry.
2. Western blotting analysis of STAT3 and phosphorylated STAT3 (p-STAT3) in FBP1-overexpressing and control A2780/SKOV3 cells. Representative data from three repeated experiments is shown.
3. Immunohistochemical staining of FBP1, STAT3 and p-STAT3 in the FBP1-overexpressing and control tissues.

**Supplementary Figure 9. FBP1 G260R mutant directly interacts with STATS and inhibits STAT3 expression and phosphorylation in ovarian cancer cells**

**(A)** Representative image of established stable cell lines showed that FBP1 G260R-overexpressing vector were constructed and stably expressed in A2780/SKOV3 cells.

**(B)** FBP1 enzymatic activity in the A2780 and SKOV3 cell lysates.

**(C)**InteraCction between FBP1 G260R and STAT3 detected by Co-immunoprecipitation assay.

**(D)** Induction of FBP1 or FBP1 G260R significantly changed the distribution and expression of STAT3 and p-STAT3 (Tyr705) protein in the nucleus and cytoplasm.

**(E)** Interaction between FBP1 G260R and STAT3 in the nucleus and cytoplasm detected by Co-immunoprecipitation assay.

**(F)** Interaction between FBP1 G260R and STAT3 by FRET-FLIM upon transient coexpression in A2780 and SKOV3 cells. FE, FRET efficiency. Asterisks indicate a statistically significant difference (**, *P* value < 0.01), according to a Student’s t test.

**(G)** Representative immunofluorescence staining (×1000) images showing that FBP1 G260R inhibited the expression of STAT3 in the cell nucleus (red). Blue dye (DAPI) indicates the nucleus.

**Supplementary Figure 10. FBP1 G260R mutant inhibits cell proliferation, glycolysis, metastasis and chemosensitivity in ovarian cancer cells.**

1. Representative images and quantitative analysis of CCK-8 Kit assay rate showed the changes of cell proliferation rate after upregulated FBP1 G260R as well as STAT3 at low glucose level.
2. Quantitative analysis of colony formation rate showed the changes of cell colonies after upregulated FBP1 G260R as well as STAT3.
3. Quantitative analysis of invaded cells showed the changes of cell invasion by using Transwell assay after upregulated FBP1 G260R as well as STAT3.
4. The glucose uptake was tested after upregulated FBP1 G260R as well as STAT3.
5. The lactate production was tested after upregulated FBP1 G260R as well as STAT3.
6. Quantitative analysis of apoptotic cells showed the changes of the cisplatin-induced apoptosis detected by flow cytometry after upregulated FBP1 G260R as well as STAT3.
7. Representative images of the relative protein expression detected by Western blotting in rescue experiment with cisplatin treatment.
8. Representative images of the relative protein expression detected by Western blotting in rescue experiment. ** *P* < 0.01.

**Supplementary Figure 11. The anti-cancer effect of FBP1 may be achieved by inhibiting the expression of STAT3.**

**(A-B)** Representative images and quantitative analysis of colony formation rate showed the changes of cell colonies after upregulated FBP1 as well as STAT3.

**(C-D)** Representative images and quantitative analysis of invaded cells showed the changes of cell invasion by using Transwell assay after upregulated FBP1 as well as STAT3.

**(E-F)** The glucose uptake, lactate production was tested after upregulated FBP1 as well as STAT3.

**(G)** Representative images of the relative protein expression detected by Western blotting in rescue experiment.

**(H)** Quantitative analysis of apoptotic cells showed the changes of the cisplatin-induced apoptosis detected by flow cytometry after upregulated FBP1 as well as STAT3.

**(I)** Representative images of the relative protein expression detected by Western blotting in rescue experiment with cisplatin treatment. * *P* < 0.05, ** *P* < 0.01.

**Supplementary Figure 12. The anti-cancer effect of FBP1 may be achieved by inhibiting the expression of STAT3 *in vivo.***

1. Representative image of nude mice bearing tumors formed by SKOV3 cells.
2. The average tumor volume with or without cisplatin treatment formed by SKOV3 cells.
3. Representative image of nude mice bearing tumors formed by A2780 cells.
4. The average tumor volume with or without cisplatin treatment formed by A2780 cells.

**Supplementary Figure 13. Tumor-suppressive properties mediated by C-MYC silencing are attenuated when FBP1 expression is abrogated.**

**(A)** Immunoblotting analysis of background expression level of FBP1 and C-MYC in four ovarian cancer cell lines.

**(B)** Immunoblotting analysis of knockdown of c-myc in A2780/SKOV3 cells.

**(C)** Representative images and quantitative analysis of colony formation rate showed the changes of cell colonies after knockdown of c-myc as well as FBP1.

**(D)** Representative images and quantitative analysis of invaded cells showed the changes of cell invasion by using Transwell assay after knockdown of c-myc as well as FBP1.

**(E-F)** The glucose uptake and lactate production was tested after silencing c-myc as well as FBP1.

**(G)** Quantitative analysis of apoptotic cells showed the changes of the cisplatin-incuced apoptosis detected by flow cytometry after knockdown of C-MYC as well as FBP1.

**(H-I)** Representative images of the relative protein expression detected by Western blotting in rescue experiment with or without cisplatin treatment. ** *P* < 0.01.
